# Streamlined Subpopulation, Subtype, and Recombination Analysis of HIV-1 Half-Genome Sequences Generated by High-Throughput Sequencing

**DOI:** 10.1128/mSphere.00551-20

**Published:** 2020-10-14

**Authors:** Bhavna Hora, Naila Gulzar, Yue Chen, Konstantinos Karagiannis, Fangping Cai, Chang Su, Krista Smith, Vahan Simonyan, Sharaf Ali Shah, Manzoor Ahmed, Ana M. Sanchez, Mars Stone, Myron S. Cohen, Thomas N. Denny, Raja Mazumder, Feng Gao

**Affiliations:** a Duke Human Vaccine Institute, Duke University Medical Center, Durham, North Carolina, USA; b Department of Biochemistry & Molecular Medicine, The George Washington School of Medicine and Health Sciences, Washington, DC, USA; c Bridge Consultants Foundation, Karachi, Pakistan; d Locus Biosciences, Morrisville, North Carolina, USA; e Blood Systems Research Institute, San Francisco, California, USA; f Department of Medicine, University of North Carolina at Chapel Hill, Chapel Hill, North Carolina, USA; g Department of Medicine, Duke University Medical Center, Durham, North Carolina, USA; h Department of Epidemiology, University of North Carolina at Chapel Hill, Chapel Hill, North Carolina, USA; i Department of Microbiology and Immunology, University of North Carolina at Chapel Hill, Chapel Hill, North Carolina, USA; University of Michigan-Ann Arbor

**Keywords:** HIV-1, genetic recombination, quasispecies, sequencing

## Abstract

High-throughput sequencing (HTS) has been widely used to characterize HIV-1 genome sequences. There are no algorithms currently that can directly determine genotype and quasispecies population using short HTS reads generated from long genome sequences without additional software. To establish a robust subpopulation, subtype, and recombination analysis workflow, we amplified the HIV-1 3′-half genome from plasma samples of 65 HIV-1-infected individuals and sequenced the entire amplicon (∼4,500 bp) by HTS. With direct analysis of raw reads using HIVE-hexahedron, we showed that 48% of samples harbored 2 to 13 subpopulations. We identified various subtypes (17 A1s, 4 Bs, 27 Cs, 6 CRF02_AGs, and 11 unique recombinant forms) and defined recombinant breakpoints of 10 recombinants. These results were validated with viral genome sequences generated by single genome sequencing (SGS) or the analysis of consensus sequence of the HTS reads. The HIVE-hexahedron workflow is more sensitive and accurate than just evaluating the consensus sequence and also more cost-effective than SGS.

**IMPORTANCE** The highly recombinogenic nature of human immunodeficiency virus type 1 (HIV-1) leads to recombination and emergence of quasispecies. It is important to reliably identify subpopulations to understand the complexity of a viral population for drug resistance surveillance and vaccine development. High-throughput sequencing (HTS) provides improved resolution over Sanger sequencing for the analysis of heterogeneous viral subpopulations. However, current methods of analysis of HTS reads are unable to fully address accurate population reconstruction. Hence, there is a dire need for a more sensitive, accurate, user-friendly, and cost-effective method to analyze viral quasispecies. For this purpose, we have improved the HIVE-hexahedron algorithm that we previously developed with *in silico* short sequences to analyze raw HTS short reads. The significance of this study is that our standalone algorithm enables a streamlined analysis of quasispecies, subtype, and recombination patterns from long HIV-1 genome regions without the need of additional sequence analysis tools. Distinct viral populations and recombination patterns identified by HIVE-hexahedron are further validated by comparison with sequences obtained by single genome sequencing (SGS).

## Abstract (precis)

The highly recombinogenic nature of human immunodeficiency virus type 1 (HIV-1) leads to recombination and emergence of quasispecies. It is important to reliably identify subpopulations to understand the complexity of a viral population for drug resistance surveillance and vaccine development. High-throughput sequencing (HTS) provides improved resolution over Sanger sequencing for the analysis of heterogeneous viral subpopulations. However, current methods of analysis of HTS reads are unable to fully address accurate population reconstruction. Hence, there is a dire need for a more sensitive, accurate, user-friendly, and cost-effective method to analyze viral quasispecies. For this purpose, we have improved the HIVE-hexahedron algorithm that we previously developed with *in silico* short sequences to analyze raw HTS short reads. The significance of this study is that our standalone algorithm enables a streamlined analysis of quasispecies, subtype, and recombination patterns from long HIV-1 genome regions without the need of additional sequence analysis tools. Distinct viral populations and recombination patterns identified by HIVE-hexahedron are further validated by comparison with sequences obtained by single genome sequencing (SGS).

## INTRODUCTION

The genome of human immunodeficiency virus type I (HIV-1) is highly variable due to the error-prone reverse transcriptase (1, 2) and short life cycle during infection (3, 4). Genetic diversity of a viral population in an infected individual increases by ∼1% annually during natural infection history (5). Thus, the viral population in a chronically infected individual is constituted of a swarm of genetically similar but distinct viruses, or quasispecies (6, 7). This high genetic variability plays an important role in escape from host immune selection pressure and development of drug resistance (8–11). The complex quasispecies nature of HIV-1 makes it difficult to analyze the viral population in each infected individual. This genetic variability results in 10 subtypes among group M viruses (https://www.hiv.lanl.gov/) (12–14). Different subtypes can have significant impacts on pathogenesis, disease progression, and drug treatment efficacy (9–11). The amino acid difference in the envelope glycoprotein (Env) can vary up to 30% (15). This extraordinary level of genetic variation poses an obstacle for development of cross-reactive vaccines and challenges for genetics-based detection assays (9–11). Due to the highly recombinogenic nature of HIV-1, over 100 circulating recombinant forms (CRFs) and many unique recombinant forms (URFs) among different subtypes have been identified (13, 16). The recombinant viruses are often detected in regions where two or more distinct subtypes are present and account for ∼20% of the global HIV-1 strains (11, 12). Since it is more difficult to detect recombinants among the viruses from the same subtype (17–19), their actual percentage in the world may be much higher.

Analysis of HIV-1 genome sequences is important for understanding viral evolution, subtype distribution, transmission, pathogenesis, transmission network, drug resistance surveillance, and vaccine development (9, 12, 15, 20–23). A large number of assays have been developed to study genetic diversity at the population and individual levels. Among them, the most widely used is the population sequencing method that directly sequences the bulk PCR products using Sanger sequencing (5, 6). While this method is simple and commonly used for subtyping and drug resistance surveillance, it only generates a consensus sequence of all viral populations in a PCR product from the sample. The final sequences often contain ambiguous bases because of complex quasispecies in the sample. When viral diversity is studied by sequencing multiple clones from a bulk PCR product, results can be seriously affected by resampling, artificial misincorporation errors, and recombinants generated during PCR (24, 25). To overcome these issues, a single genome sequencing (SGS) method was developed by PCR amplification of single viral genomes in individual reactions (26, 27). SGS can reliably study the viral populations and has played an important role in analysis of linked drug resistance mutations in the same viral genome (26) and determination of transmitted/founder viruses that establish clinical HIV-1 infection (27, 28). But it is labor-intensive, time-consuming, and costly.

High-throughput sequencing (HTS) that produces massively parallel sequences is now being used to study complex genome populations (29–33). HTS provides improved resolution over Sanger sequencing for identification and analysis of heterogeneous viral subpopulations (31, 34). Initially, the high cost of HTS was inhibitory for studying a quasispecies population in a sample. However, recently using sequence identifiers and tags, it provides unprecedented promise for large-scale sequencing of the viral population in a simpler, cost-effective, and time-efficient manner (35–38). Two kinds of HTS methods have been developed: long read (PacBio and Nanopore) and short read (Illumina, Ion Torrent, and 454). Though PacBio and Nanopore sequencing technologies have the advantage of longer read lengths (up to 900 kb), they have the disadvantage of lower throughput, higher error rate, and higher cost per base (39–41). Among HTS methods, Illumina is the most commonly used with an impressive increase in throughput and the lowest per-base cost (39–42). Currently, HTS has been widely used to analyze viral quasispecies for small genomic regions (<500 bp). Minority variants present at ≥1% frequency in a sample can be characterized by HTS when sufficiently large numbers of templates are analyzed (33, 43). However, when long-range PCR products (>4,000 bp) are analyzed, it is difficult to reliably identify subpopulations to understand the complexity of a quasispecies because short sequence reads cannot be reliably assembled into full-length consensus sequences for each subpopulation in the sample.

Specialized sequence analysis is required to study the genetic diversity using HTS data. With advancement of analytical techniques, many algorithms have become available to analyze HTS short-read data, but accurate population reconstruction is still elusive. Despite significant improvements in analytical approaches, there has not been significant effort to address this question, and each of the current approaches has different limitations. For example, traditional algorithms like ShoRah (44) can align only a limited number of reads (up to tens of thousands) and call haplotypes based on the center of clusters. ViSpA (45) and QuRe (46) were designed to analyze reads generated by pyrosequencing and focus on identifying insertions and deletions as well as errors in homopolymeric regions. PredictHaplo (47) and HaploClique (48) can analyze data from methods other than pyrosequencing but still are limited in the number of reads that can be analyzed.

High-performance Integrated Virtual Environment (HIVE) is a robust cloud-based infrastructure designed to handle HTS data (49, 50). It provides secure web access to registered users to store, retrieve, annotate, and compute HTS data and visualize the outcome of computations on a user-friendly interface (49, 50). HIVE-hexahedron is a novel deterministic algorithm that can overcome the aforementioned limitations and process the HTS data at a much larger scale than before (32). This algorithm, when used with *in silico* reads, showed an improved performance in sequence reconstruction and identification of discrete populations in heterogeneous samples (51).

Targeted small regions (<600 bp) of HIV-1 genomes have been widely used to study viral populations in samples using short-read HTS methods (33, 43). However, because of the high levels of genetic variation and recombinogenic nature of HIV-1, such short sequences do not reveal the real quasispecies nature of HIV-1 in a given sample (52–54). When half or whole genomes are sequenced by HTS, the quasispecies cannot be determined with the current computational tools (44, 55–58). In addition, subtyping and recombination analyses of sequences generated by HTS from long sequences (>4,000 bp) need to be performed using additional conventional software (46, 47). To be able to simultaneously determine viral subpopulations, subtypes, and recombination patterns in a sample with HTS short reads on a single platform, we developed a new HIVE-hexahedron algorithm by incorporating a Nearest Neighbor pipeline and introduced advanced parameters in HIVE-hexagon (aligner). We tested the pipeline by analyzing HTS short reads from HIV-1 3′-half genome sequences (∼4,500 bp) generated from 65 chronically infected individuals. More importantly, results from HIVE-hexahedron were validated by the consensus sequences generated by the commonly used Geneious software for all samples and multiple individual viral genome sequences generated by SGS from seven samples. Our results demonstrate that HIVE-hexahedron can simultaneously determine subpopulations, subtypes, coinfections, and recombination of a viral population by analyzing long-range sequences generated by short HTS reads without exporting sequences for additional analysis using other computational tools.

## RESULTS

### Polymorphic sites in a viral population are not resolvable in the consensus sequence generated by Geneious.

The raw paired-end HTS reads from each sample were loaded to the Geneious assembler and mapped to 3′-half genome sequence from reference HXB2 using medium/fast sensitivity and five-time iterations of the assemblies with default parameters. After the reads were mapped to the reference, the majority of the samples (52 of 65) had <2% of unused reads while the rest had 2.22% to 21% unused reads. The mean coverage was between 360 and 63,195 at any nucleotide position. The assembled reads resulted in one contig, and the consensus of the contig was generated. Phylogenetic analysis of the consensus sequences from 65 samples showed 17 subtype A1s (26.15%), 4 subtype Bs (6.15%), 27 subtype Cs (41.5%), 7 CRF02_AGs (10.76%), and 10 URFs (15.38%) (Table 1). Since the consensus sequence generated by the Geneious assembler is an aggregate of all variants at each site within the population, ambiguous bases were detected in 43 sequences (Table 1). Among them, 11 sequences had more than 50 ambiguous bases and as many as 217 ambiguous bases were found in one sequence (707010585), suggesting the presence of quasispecies in samples with many ambiguous bases.

**TABLE 1:** Diversity of the viral population in samples determined by HIVE and Geneious

Sample ID	Viral load (copies/ml)	Subtype	No. of ambiguous bases detected by Geneious	No. of viral populations detected by HIVE
700010501	31,875	B	0	1
700010516	10,310	B	0	1
702010118	32,081	C	0	1
702010322	57,901	C	0	1
702010675	146,346	C	0	1
704010566	26,700	C	0	1
705010303	26,833	C	0	1
705010661	31,081	C	0	1
705010801	4,704	C	0	1
706010391	271,000	C	0	1
707010277	97,122	URF_A1C	0	1
PK005	610	CRF02_AG	0	1
PK010	1,250	C	0	1
PK011	8,750	URF_A102	0	1
PK021	93,500	A1	0	1
PK025	26,900	CRF02_AG	0	1
PK032	6,400	CRF02_AG	0	1
PK034	4,665	A1	0	1
702010350	85,381	C	0	2
703010619	29,059	C	0	1
703010632	43,600	C	0	2
PK016	13,000	A1	0	1
706010375	119,000	C	0	1
705010366	15,329	C	0	1
PK026	5,350	A1	1	1
703010539	97,857	C	2	1
703011871	150,182	C	3	1
704010715	319,000	C	4	1
703010523	88,878	C	5	1
PK036	96,500	A1	5	1
PK039	60,000	CRF02_AG	6	2
PK009	173,000	C	7	1
705010381	21,352	C	8	2
PK007	106,000	A1	8	2
700010329	11,014	B	11	1
PK004	464,000	A1	11	4
705010614	98,700	C	12	1
PK027	111,500	A1	15	4
705010645	425,760	C	16	1
PK038	456,000	CRF02_AG	16	3
707010038	17,213	A1	16	3
703010234	183,452	C	17	1
PK019	760,000	CRF02_AG	18	3
705010474	40,497	C	20	1
PK031	225,500	A1	20	3
PK001	70,000	A1	23	6
PK020	208,000	A1	24	5
PK023	65,500	URF_A102	27	4
PK035	461,500	CRF02_AG	27	5
PK002	488,000	A1	34	9
PK040	111,000	A1	38	13
700010260	74,588	B	42	2
PK015	300,000	URF_A102	42	6
704010486	253,000	C	48	3
PK018	88,500	A1	57	6
705010699	46,057	C	61	2
PK008	625,000	URF_A102	61	5
PK006	710,000	URF_A102	67	8
PK013	193,000	A1	69	7
PK033	425,500	URF_A102	71	7
PK017	119,000	A1	80	5
702010133	591,344	C	81	3
PK003	13,600	URF_A102	100	3
707010134	15,208	URF_CD	166	3
707010585	79,367	URF_A1C	217	5

### Identification of distinct HIV-1 subpopulations in the same samples by HIVE-hexahedron.

**FIG 1:**
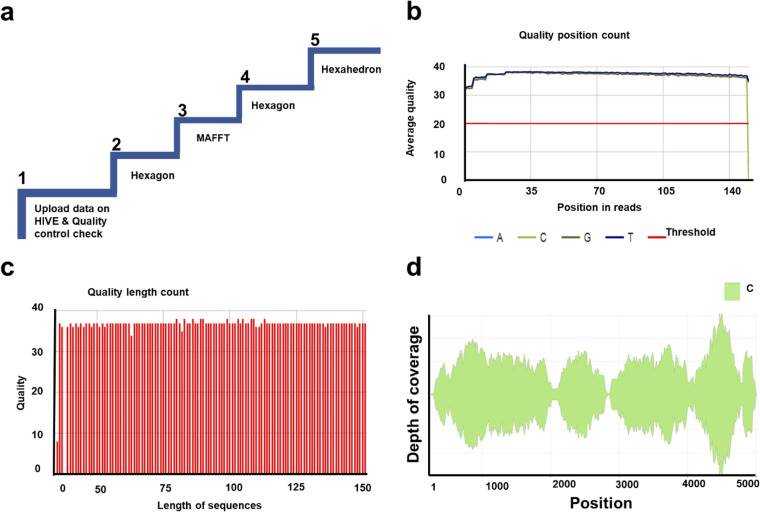
HIVE pipeline for subtype and quasispecies analysis of HIV-1 3′-half genome sequences generated by HTS. The sample 704010715 is used for illustration of the steps involved in the pipeline. (a) The overview of HIVE analysis pipeline involves five steps. Step 1, raw sequencing reads are uploaded on the High-performance Integrated Virtual Environment (HIVE) platform and concurrently quality control graphs are generated for each read file. Step 2, identification of nearest neighbor references using HIVE-hexagon alignment tool. Each paired-end read file is aligned with a genotype reference set of 20 consensus sequences for 8 subtypes (A1, A2, B, C, D, F1, G, and H) and 10 major circulating recombinant forms (CRFs) from the Los Alamos HIV-1 sequence database. The references with >10,000 RPKM are selected as nearest neighbors for further analysis. Step 3, a multiple sequence alignment of nearest neighbor references is performed using MAFFT in case there is more than one reference with >10,000 RPKM. Step 4, read sequences are aligned against the nearest neighbor reference(s) using HIVE-hexagon. Step 5, subtype and quasispecies analysis using the HIVE-hexahedron tool. (b) Quality position count graph depicts the average quality of reads per position with a Phred score of 30 as threshold. (c) Quality length count graph depicts quality of sequences at different lengths. (d) The results of HIVE-hexahedron analysis are visualized using an interactive Sankey diagram. The color of the Sankey diagram indicates the subtype (in this case, subtype C), the length of the genome is represented along the *x* axis, and thickness represented along the *y* axis reflects the depth of coverage. Consensus sequences, alignment, composition, and summary of the selected or all viral populations are downloadable for further analysis. To generate consistent and reliable results, the tool allows some postprocessing to filter out the viral populations with length of less than 500 bases, average coverage less than 50, and average variations less than 5 bases. The narrower regions in the Sankey diagram at position 2,000 are due to large insertions and/or deletions at the sites. HIVE-hexahedron filters out those reads which have big insertions/deletions toward the ends. This results in low depth of coverage at those regions.

**FIG 2:**
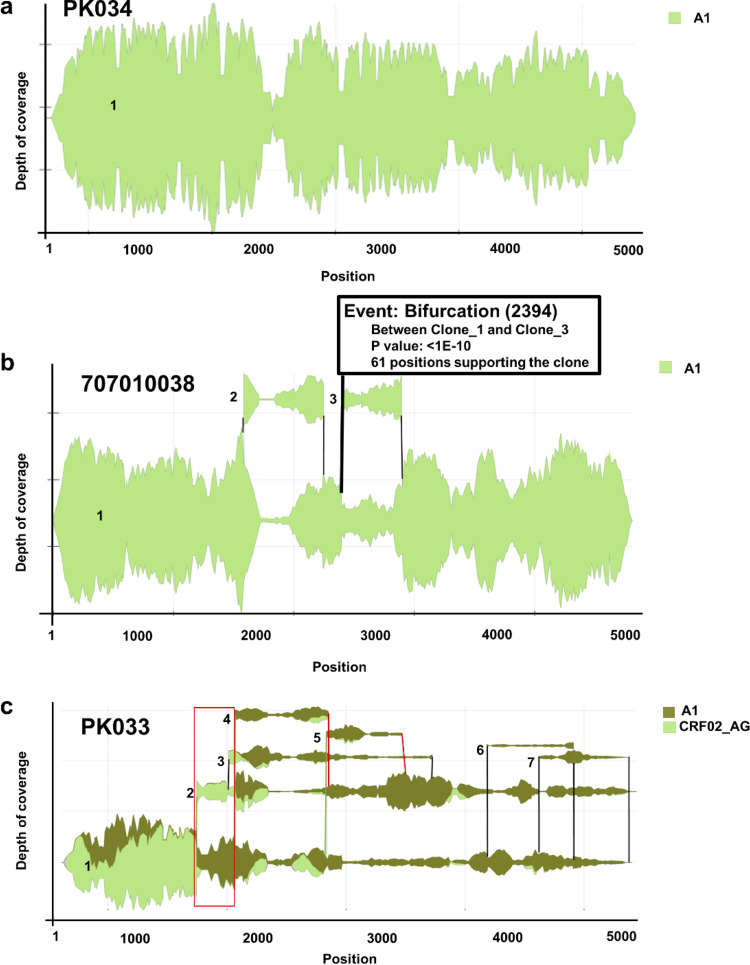
Identification of distinct viral populations by HIVE-hexahedron. (a) The analysis of HTS reads from PK034 using the Sankey diagram tool indicates that there is one nearest neighbor (subtype A1) indicated by light green color. A single horizontal line represents a single viral population in PK034. (b) The analysis of HTS reads from 707010038 shows one nearest neighbor (subtype A1) with three distinct viral populations: the main contig (1) spanning the 3′-half HIV-1 genome and two smaller contigs (2 and 3) representing partial 3′-half HIV-1 genome. A small pop-up window over contig 3 shows the details about the number of sites supporting the population and the *P* value, when a cursor is placed over a bifurcation or merging lines. (c) The analysis of HTS reads from PK033 indicates two nearest neighbors (subtype A1 and CRF02_AG). The sequence reads are resolved into seven contigs, with the major contig 1 spanning the 3′-half HIV-1 genome and six smaller contigs (2 to 7). Potential recombination patterns between two different neighbor reference sequences are indicated by different shades of green. The black vertical lines mark bifurcation from and merging into the same parent contigs. Gray vertical lines mark bifurcation position from the parent contigs, and red vertical lines mark the merging into nonparent contigs. The region that is represented by CRF02_AG (in contig 2; light green) and A1 (in contig 1; dark green) is indicated by a red box.

We previously developed a HIVE-hexahedron algorithm and determined subpopulations using simulated *in silico* sequence data sets (32). In order to investigate if the ambiguous bases in the consensus sequences generated by Geneious indicate the presence of viral populations in those samples, we analyzed the same sequence data sets using HIVE-hexahedron. Several modifications were made in the original algorithm to better define viral subpopulations in the real HTS data from 65 HIV-1 samples (see Materials and Methods). Five steps were followed to determine the subtype(s) and the number of subpopulations in the samples (Fig. 1a). First, the quality of HTS reads from each sample was inspected using quality control graphs for “Quality length counts” (Fig. 1b) and “Quality position count” (Fig. 1c) generated in HIVE. All the reads selected for subpopulation analysis had quality length count and quality position count higher than a 30 Phred score. The first alignment step was to select the nearest neighbor references (with ≥10,000 reads per kilobase of transcript per million [RPKM]) for optimal assembling. Each read from a sample was mapped to the best-scored reference out of the reference set using the alignment tool HIVE-hexagon (59). The tool uses some advanced features for trimming loosely aligned ends. This is also a primary step to determine subtypes of the viral sequence. The second alignment was performed on the reads per sample against their nearest neighbor reference(s). If more than one nearest neighbor reference was identified in a sample, a multiple sequence alignment (MSA) of the nearest neighbor references was done using MAFFT (60). The results of the second alignment were remapped with a common coordinate system generated by MAFFT using HIVE-hexahedron. The multiple sequence alignments of the nearest neighbor references were not imperative to the HIVE-hexahedron. However, multiple references covering divergent HIV-1 sequences will provide a better platform for reference-assisted *de novo* assembly of HIV-1 populations and identify coinfections by different subtypes or recombinant viruses. The results were further improved by postprocessing steps (see Materials and Methods) and visualized using Sankey diagrams (Fig. 1d). HIVE-hexahedron displays subpopulation analysis results in the form of interactive Sankey diagrams, which exhibit the coverage per position, subtype(s) of each viral subpopulation, and number of variations that support each subpopulation. Each band in the Sankey diagram represents a separately reconstructed subpopulation, with vertical gray and red lines representing bifurcation and merging events, respectively (Fig. 2). The bifurcation and merging events depict where a subpopulation is detected and where it ends, respectively, relative to the coordinates of multiple sequence alignment (MSA) or the coordinates of resolved sequences. A population is considered major if it covers the entire length of the amplicon. Minor subpopulations are short contigs that bifurcate from the parent population and have at least five mutations in a 500-bp region (1%) compared to its parent. They have an average depth of coverage of 50 and minimum length of 500 bp. The Sankey plots provide a visualization of the reads that support the subpopulation detection. The algorithm resolves the subpopulation and calculates the depth of coverage for each population at each position. Thus, the width of each Sankey diagram is an indicator of the relative abundance at each position. As part of the interface, the user can postcomputationally retrieve the relative abundance of the inferred globally reconstructed sequences for all populations at any given positions.

An example of the major and minor viral subpopulations observed in three samples using Sankey diagrams is shown in Fig. 2. Only one population was detected in PK034 (Fig. 2a), indicating a relatively homogenous viral population. The light green color of the Sankey diagram represents the subtype of the virus, in this case subtype A1. The width of the Sankey diagram represents the depth of coverage per position along the *x* axis. Three subpopulations were detected in 707010038: one major and two minor (Fig. 2b). All trajectories following the bifurcation and merging events are assemblies representing possible different subpopulations (32). The subpopulations 2 and 3 differed from the major population by 1.6% and 6.2%, respectively. The detection of three subpopulations in 707010038 also explains the unresolved 16 ambiguous bases detected by Geneious. In PK033, eight subpopulations were identified: one major and seven minor (Fig. 2c). In addition to multiple subpopulations, two nearest neighbor references were identified by HIVE-hexahedron, and they are shown in light green (CRF02_AG) and dark green (A1) in Sankey diagrams (Fig. 2c). The genetic differences among consensus sequences of all viral subpopulations were high (3.63% to 11.8%) and this also explains why there were 71 ambiguous bases that could not be resolved by Geneious (Table 1).

**FIG 3:**
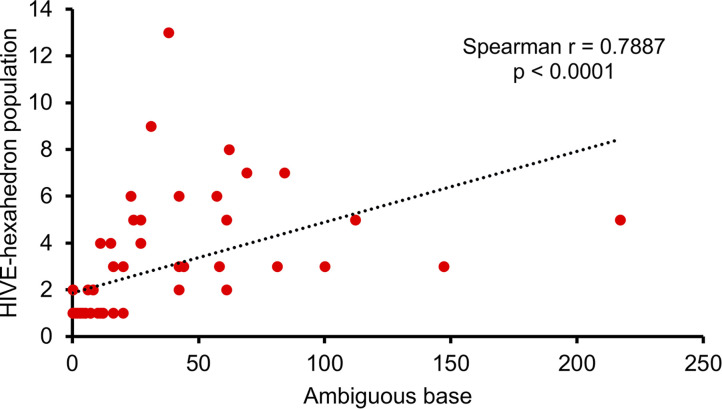
Association of the numbers of viral populations determined by HIVE-hexahedron and the number of ambiguous bases in Geneious-derived consensus sequences. The scatterplots were generated with a simple linear regression analysis of the numbers of viral populations determined by HIVE-hexahedron and ambiguous bases in Geneious-derived consensus sequences from 65 samples.

Sequence analysis using HIVE-hexahedron showed that 47.69% (31/65) of samples harbored subpopulations. Twenty-four samples (36.92%) had 2 to 5 subpopulations, and six samples (9.23%) had 6 to 9 subpopulations, while one sample (1.53%) had up to 13 subpopulations (Table 1; see also Fig. S1 in the supplemental material). Thirty-four samples that did not have detectable subpopulations by HIVE-hexahedron had 20 or fewer ambiguous bases detected by Geneious. All 22 samples with no ambiguous bases by Geneious and the majority (28/30; 93.33%) of the samples with ≤5 ambiguous bases had only one detectable subpopulation by HIVE-hexahedron. Conversely, the majority (29/35; 82.85%) of the samples with ≥5 ambiguous bases had more than one detectable subpopulation (Table 1 and Fig. S1). All 20 samples with >20 ambiguous bases had ≥2 subpopulations. The numbers of ambiguous bases identified by Geneious were significantly correlated with the numbers of subpopulations detected in samples by HIVE-hexahedron (nonparametric Spearman correlation *r* = 0.8015; *P* < 0.0001) (Fig. 3).

10.1128/mSphere.00551-20.1 FIG S1 Detection of viral subpopulations in chronic HIV-1 infection samples. HTS reads from 65 samples were individually analyzed using the Sankey diagram tool. Single color indicates the presence of only one subtype, and two shades of green represent the presence of two different subtypes or CRFs. The length of the genome is represented along the *x* axis, and thickness represented along the *y* axis reflects the depth of coverage. Consensus sequences, alignment, composition, and summary of the selected or all viral populations are downloadable for further analysis. The major contig spans the entire length of the 3′-half genome, and the minor contigs are projected as regions that bifurcate from the major contig and merge back. Download FIG S1, PDF file, 2.1 MB. Copyright © 2020 Hora et al. 2020 Hora et al. This content is distributed under the terms of the Creative Commons Attribution 4.0 International license.

### Determination of HIV-1 subtypes.

**FIG 4:**
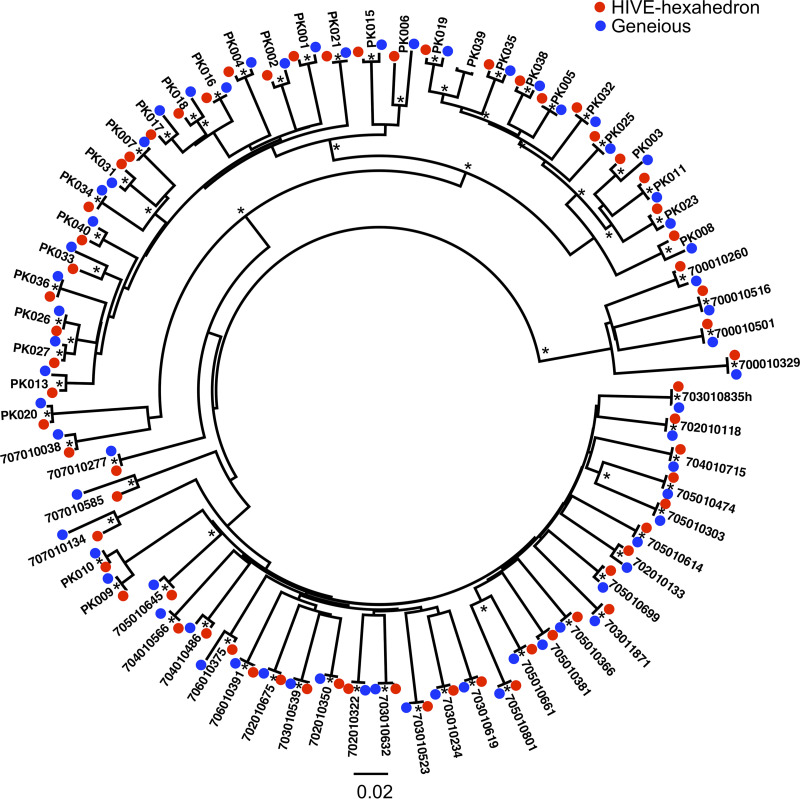
Phylogenetic tree analysis of sequences assembled from HTS reads by Geneious and HIVE. The consensus sequences of contigs assembled using Geneious (blue) and the consensus sequences for the major full-length viral population assembled using HIVE-hexahedron (red) from all samples were aligned together using CLUSTAL W. The phylogenetic tree was constructed using the neighbor-joining method and the Kimura two-parameter model. The scale bar represents 0.02 nucleotide substitutions per site. Asterisks indicate bootstrap values for the knots that are supported in 95% or more of replicates (out of 1,000).

One of the unique advantages of HIVE-hexahedron is that multiple sequences can be simultaneously used as references. This allows it to determine which HIV-1 subtype reference(s) the test sequences are most similar to and thus determine the subtypes of the viral sequences. Due to high variability among viral sequences in each subtype, we used subtype consensus sequences from the Los Alamos HIV sequence database to better define subtypes of the samples. This set of 20 consensus sequences contains eight subtypes (A1, A2, B, C, D, F1, G, and H) and 10 CRFs (which cover all subtypes detected in our samples by analyzing the Geneious-derived sequences) and two outlier controls (group O and SIVcpz). The raw read sequences from each sample were mapped to this set of 20 HIV-1 references using HIVE-hexahedron. The specific algorithm in HIVE-hexahedron was used to select the best-scoring reference for each read sequence in a sample. Thus, the first alignment step in HIVE-hexagon can determine subtype references that are the nearest neighbors for the reads (Fig. 1d). This is defined by the RPKM values greater than 10,000. When more than one nearest neighbor was identified by HIVE-hexahedron, an additional alignment step(s) of the reads was carried out with the other nearest neighbor(s). The analysis of the HTS data from all 65 samples by HIVE-hexahedron showed similar subtyping results as determined by Geneious (Table 1 and Fig. S1). Phylogenetic tree analysis of the major contig consensus sequences generated from HIVE-hexahedron and Geneious-derived consensus sequences from the same sample showed that both sequences from the same sample always clustered together (Fig. 4). While the differences were generally small between the sequences generated by HIVE and Geneious from the same viruses, larger genetic distances (>0.4%) were observed for 15 viruses (707010585, 707010134, 704010486, 705010699, 702010133, 706010375, PK002, PK003, PK006, PK008, PK013, PK017, PK018, PK033, and PK040). Examination of these sequences showed that there were many ambiguous bases in the sequences generated by Geneious in all but one sample (706010375). This suggested that higher genetic differences between sequences generated by HIVE and Geneious were caused by more divergent viral populations.

### Detection of different viruses in the same sample.

More than one virus can be frequently detected in an infected individual (27, 28, 61, 62). This can often result in generation of recombinants between the different subtype viruses in such infected individuals. Thus, it is important to know how frequently individuals are infected by two distinct viruses. Although analysis of 65 viruses by HIVE-hexahedron did not identify coinfections with two or more subtypes that differ from each other across the entire 3′-half genome (Fig. S1), regions that were clearly distinct between A1 and CRF02_AG were observed in two viruses, PK033 and PK006 (Fig. 2c and Fig. S1). The distributions of raw reads from CRF02_AG (light green) and subtype A1 (dark green) varied significantly among different contigs (Fig. S2 and S3), suggesting that viruses at these regions were derived from different subtypes. Phylogenetic tree analysis of these region sequences demonstrated that they clustered with CRF02_AG and subtype A1 separately (Fig. S2 and S3). These results showed that distinct viral populations were present in these samples. Although they were different from each other at part of the 3′-half genome, different viral populations were not identified by Geneious.

10.1128/mSphere.00551-20.2 FIG S2 Detection of distinct subtypes at a region (nt 6013 to 6254) of the PK033 genome. (a) Sankey diagram of assembled PK033 HTS data by HIVE identified two nearest neighbors: subtype A1 (dark green) and CRF02_AG (light green). The distributions of raw reads from CRF02_AG (light green) and subtype A1 (dark green) varied significantly between contigs 1 and 2 (indicated by red box). (b) Phylogenetic tree analysis of discordant region sequences. The partial sequences in contigs 1 and 2 (indicated by red box) clustered with subtype A and CRF02_AG sequences separately. Download FIG S2, PDF file, 0.2 MB. Copyright © 2020 Hora et al. 2020 Hora et al. This content is distributed under the terms of the Creative Commons Attribution 4.0 International license.

10.1128/mSphere.00551-20.3 FIG S3 Recombination breakpoint analysis of PK006. (a) Sankey diagram of assembled PK006 HTS data by HIVE identified two nearest neighbors: subtype A1 (dark green) and CRF02_AG (light green). The potential recombination breakpoints (based on the positions in HXB2) were exported for the populations on the HIVE platform and are marked on the contigs on the Sankey diagram. (b) Recombination breakpoints in all the contig sequences were directly determined by HIVE and are schematically presented using the Recombinant HIV-1 Drawing Tool. Recombination breakpoints are indicated at the top of the contigs. (c) Recombination breakpoints of the consensus sequence determined by Geneious were analyzed using jpHHM and Simplot and schematically presented using the Recombinant HIV-1 Drawing Tool. Recombination breakpoints are indicated based on the coordinates in the HXB2 genome. The different recombination patterns and high levels of genetic diversity (1.9% to 10.4%, the levels between different subtypes) among HIVE contigs (1 and 5 to 8) at the 3′ end of the genome suggest the presence of multiple different viruses in the sample that could not be resolved by Geneious and caused differences in the recombination breakpoints. (d) Phylogenetic tree analysis of discordant region sequences. The partial sequences in contigs 1, 5, and 6 and contigs 1, 5, 7, and 8 (indicated by red boxes) clustered with subtype A and CRF02_AG sequences separately. Download FIG S3, PDF file, 0.3 MB. Copyright © 2020 Hora et al. 2020 Hora et al. This content is distributed under the terms of the Creative Commons Attribution 4.0 International license.

### Detection of recombinant sequences.

**FIG 5:**
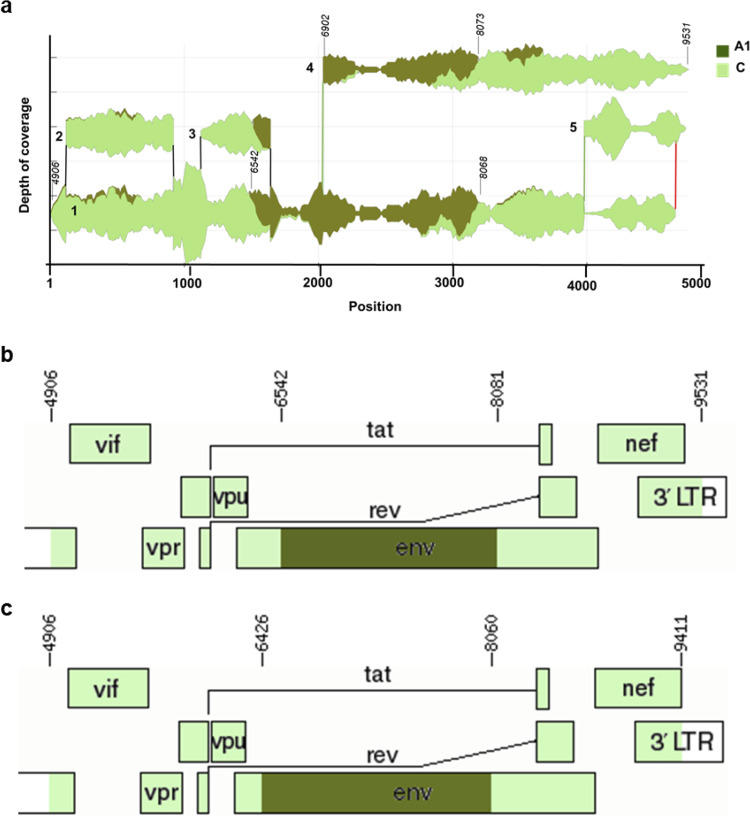
Direct identification of recombination patterns using HIVE. (a) Viral populations and recombination patterns were detected by analyzing 707010585 HTS data using HIVE-hexahedron. The analysis identified two nearest neighbors: subtype A1 (dark green) and subtype C (light green). The potential recombination breakpoints (based on the positions in HXB2) were exported for the populations on the HIVE platform and are marked on the viral subpopulations on the Sankey diagram. (b) Recombination breakpoints of the major population sequence determined by HIVE are schematically presented using the Recombinant HIV-1 Drawing Tool. LTR, long terminal repeat. (c) Recombination breakpoints of the consensus sequence determined by Geneious were analyzed using jpHHM and Simplot and are schematically presented using Recombinant HIV-1 Drawing Tool. Breakpoints are indicated based on the coordinates in the HXB2 genome.

When the HTS read sequences were from different subtypes or CRFs, they could be correctly defined by aligning to different nearest neighbor references in HIVE-hexahedron (Fig. 2 and Fig. S1). This should allow detection of recombinant genomes and define recombination breakpoints between different subtypes and/or CRFs using HIVE-hexahedron. For example, both subtype A1 and C read sequences were detected in 707010585 and aligned to corresponding reference sequences, shown in the Sankey diagram. The recombination pattern of 707010585 was visualized by the Sankey diagram (Fig. 5a). It contained three recombinant regions: subtype C (*vif-tat-rev-vpu-env* beginning; nucleotide [nt] 4906 to 6542 [based on positions in HXB2]), subtype A1 (middle part of *env*; nt 6543 to 8081), and subtype C (end of *env-tat-rev-nef*; nt 8082 to 9531), as schematically shown in Fig. 5b. Analysis of the 707010585 consensus sequence generated by Geneious, using jpHHM and Simplot, showed similar recombination breakpoints (Fig. 5c). Analysis of all consensus sequences generated by Geneious identified 10 URF viruses (6 CRF02_AG sequences were excluded [Table 1]). Analysis of the same 10 sequences by HIVE-hexahedron confirmed recombinant genomes (Table 1 and Fig. S1). The recombination breakpoints in the major contig sequences identified by HIVE-hexahedron were very similar to those determined by analyzing the consensus sequences generated by Geneious in nine viruses (Table 2). However, recombination breakpoints between the major population determined by HIVE-hexahedron and the Geneious-derived sequences were different in PK006. In the last part of the 3′-half genome of PK006, the Geneious-derived sequence showed a recombination pattern as CRF02-A1-CRF02, while the HIVE-hexahedron-derived major population sequence had a CRF02-A1-CRF02-A1 pattern (Fig. S3). When other partial minor subpopulation sequences were included for analysis, the recombination pattern at the first part of this region was shared between Geneious-derived sequences and HIVE-hexahedron-derived sequences for minor subpopulations 5 and 6 (Fig. S3b). The recombination pattern at the last part of this region was shared between Geneious-derived sequences and HIVE-hexahedron-derived sequences for minor subpopulations 5, 7, and 8 (Fig. S3b). Phylogenetic tree analysis of the region among all overlapping HIVE-derived contig sequences showed that sequences from contigs 5, 6, 7, and 8 clustered together with the Geneious-derived sequence, while the major contig 1 sequence branched out separately for the two regions (Fig. S3d). These results demonstrate that different viral populations that contain different recombinant patterns in a sample can be reliably detected by HIVE-hexahedron while the Geneious-derived consensus sequences of the overall viral population can detect only one of two or more different subpopulations in the sample.

**TABLE 2:** Comparison of recombination breakpoints determined by HIVE-hexahedron and Geneious-derived sequences^*a*^

Virus	HIVE-hexahedron		Geneious	
	Region	Subtype	Region	Subtype
707010585	4906–6541	C	4906–6425	C
	6542–8080	A1	6426–8059	A1
	8081–9531	C	8060–9411	C

707010134	4906–8321	C	4906–8274	C
	8322–8864	D	8275–8764	D
	8865–9621	C	8765–9392	C

PK-011	4906–8777	CRF02_AG	4906–8760	CRF02_AG
	8778–9591	A1	8761–9603	A1

PK003	4932–8920	CRF02_AG	4906–8784	CRF02_AG
	8921–9624	A1	8785–9606	A1

PK033	4906–6037	CRF02_AG	4906–6162	CRF02_AG
	6038–9608	A1	6163–9606	A1

PK008	4906–6033	A1	4906–6056	A1
	6034–7398	CRF02_AG	6056–7680	CRF02_AG
	7399–8067	A1	7681–7929	A1
	8068–8388	CRF02_AG	7930–8330	CRF02_AG
	8389–9238	A1	8331–9279	A1
	9239–9624	CRF02_AG	9280–9604	CRF02_AG

PK015	4904–6243	CRF02_AG	4906–6127	CRF02_AG
	6244–9611	A1	6128–9606	A1

707010277	4906–7025	C	4906–7072	C
	7026–8457	A1	7073–8365	A1
	8458–8776	C	8366–8672	C
	8777–9587	A1	8673–9526	A1

PK023	4907–6256	CRF02_AG	4906–6154	CRF02_AG
	6257–6473	A1	6155–6553	A1
	6474–8846	CRF02_AG	6554–8801	CRF02_AG
	8847–9624	A1	8802–9605	A1

PK006	4905–6252	CRF02_AG	4906–6192	CRF02_AG
	6253–7577	A1	6193–7516	A1
	7578–8474	CRF02_AG	7577–7876	CRF02_AG
	8475–8852	A1	7877–8176	A1
			8177–8965	CRF02_AG
	8853–9624	CRF02_AG	8966–9606	A1

a The shaded cells represent conflicting recombination patterns between Geneious and HIVE-derived sequences (Fig. S3).

### Subpopulation sequences identified by HIVE-hexahedron are confirmed by single genome sequencing.

**FIG 6:**
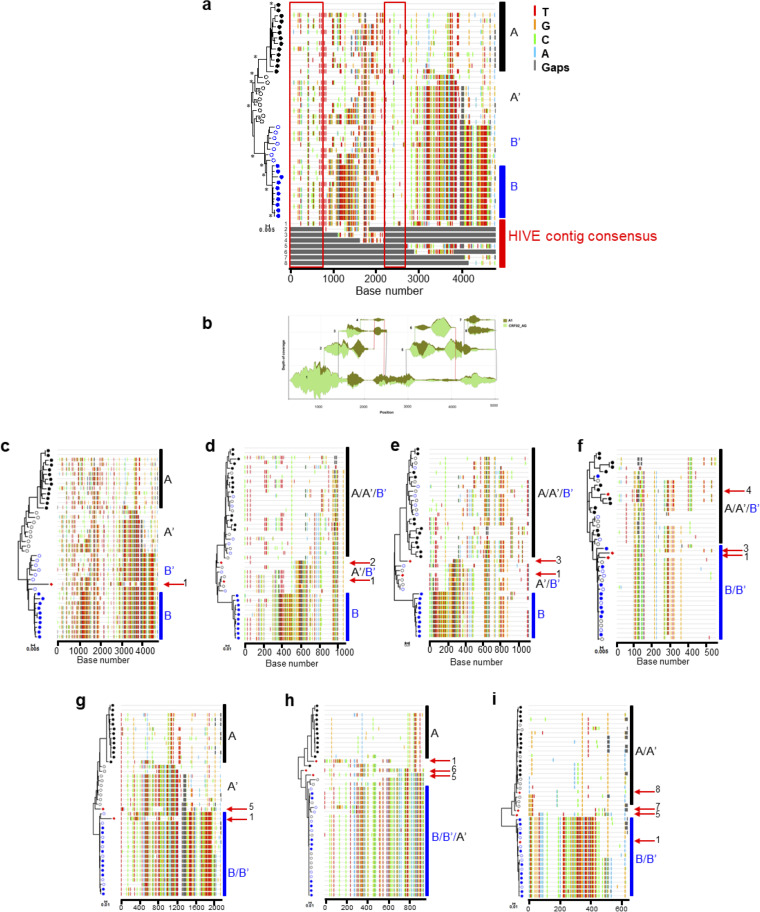
Distinct viral populations determined by HIVE are validated by analysis of SGS-derived sequences. (a) Phylogenetic tree analysis and Highlighter plot of SGS-derived sequences from PK006 identified two main viral populations (A and B; solid circles) and recombinant sequences that are either more A-like (A′) or B-like (B′) (open circles). Phylogenetic trees were constructed using the neighbor-joining method and Kimura 2-parameter model. Highlighter plots show nucleotide substitutions compared to the SGS-derived sequence at the top and color-coded nucleotide substitutions. Each SGS-derived sequence is denoted by a symbol and corresponds to the horizontal lines on the Highlighter plot. The contig consensus sequences from HIVE-hexahedron analysis are included in the Highlighter plot for comparison. Two regions that are similar among all compared sequences (red boxes) were represented only in the main contig 1. (b) The Sankey diagram showed eight contigs: one major contig spanning the entire length of the 3′-half genome (contig 1) and seven smaller contigs (contigs 2 to 8). (c to i) Phylogenetic tree and Highlighter plot analysis of complete 3′-half genome sequences (c) and partial sequences (d to i). The relationship between HIVE-hexahedron contig sequences (red arrows) and SGS-derived sequences that overlap each other was determined individually by phylogenetic trees and Highlighter plots.

One key question is whether subpopulation sequences determined by HIVE-hexahedron are indicative of viral subpopulations in real samples. To accurately characterize the viral population in a sample, we obtained complete 3′-half genome sequences by SGS using the same PCR primers used for HTS. Since the viral genomes are individually amplified and the amplicons are directly sequenced in bulk, the SGS-derived sequences are not affected by PCR-mediated misincorporation, recombination, and resampling errors (25–27). Thus, the viral population in a sample can be acurately characterized by the SGS method. Thirty-nine complete 3′-half genome sequences were obtained from PK006 by SGS (Table 3). Phylogenetic tree and Highlighter plot analyses showed that the virus had two subpopulations (A and B) and various recombinants between them (Fig. 6a).

**TABLE 3:** Number of subpopulations identified in each sample by HIVE and SGS methods

Sample	SGS		HIVE
	No. of sequences	No. of populations	No. of populations
PK006	39	3	8
707010134	54	3	3
707010038	21	1	1
PK038	34	2	3
707010585	26	4	5
PK018	53	3	5
PK013	59	2	7

Recombinant sequences could be further classified into two groups; A-like recombinants and B-like recombinants. HIVE-hexahedron analysis identified one major complete population (contig 1) and eight minor short subpopulations (contigs 2 to 8 [Fig. 6b]). To investigate if those subpopulation sequences represented the actual viral populations in the sample, the consensus sequences of all subpopulations determined by HIVE-hexahedron were exported and aligned together with all SGS sequences (Fig. 6a). We then performed phylogenetic tree and Highlighter plot analyses for each overlapping region to determine the genetic relationship between HIVE-hexahedron-derived subpopulation sequences and the SGS sequences. A longer subpopulation consensus sequence(s) was included whenever the overlapping regions covered the entire short subpopulation consensus sequence.

When the major complete consensus sequence (4,719 bp) was analyzed, it clustered with subpopulation B and B-like recombinant sequences but was different from those sequences (Fig. 6c). Thus, the major population sequence represented only subpopulation B and B-like recombinant sequences (about half of the viral population). At the regions where subpopulations 2 and 3 overlapped the major population 1, some of the SGS (A′ and B′) sequences formed a distinct cluster in the middle of the trees (Fig. 6d and e). Subpopulations 1 and 2 or 1 and 3 clustered with this group of sequences more closely than other sequences. At a region where resolution among all sequences was limited, subpopulation sequence 4 was highly similar to two of the subpopulation A sequences while subpopulation 1 and 3 sequences clustered within a clade consisting of population B, A′, and B′ sequences (Fig. 6f). Subpopulation 5 sequence clustered tightly with the population A′ sequences while the major population 1 sequence clustered within a clade consisting of population B and B′ sequences (Fig. 6g). At the region where subpopulation 5 and 6 sequences overlapped, SGS sequences formed two main clades. Interestingly, all three subpopulation sequences (1, 5, and 6) together with one of the SGS sequences represented distinct recombinant sequences between two main populations (Fig. 6h). At the end of the 3′-half genome, four subpopulations (1, 5, 7, and 8) overlapped each other (Fig. 6b). In this region, the SGS sequences formed two clades (A/A′ and B/B′). While HIVE contig 1 sequence clustered with B/B′ sequences, HIVE contig 5, 7, and 8 sequences clustered with A/A′ sequences (Fig. 6i). In fact, these A/A′ sequences and HIVE contig 5, 7, and 8 sequences were subtype A1 while the B/B′ sequences and HIVE contig 1 sequence were CRF02_AG (Table 4 and Fig. S3d). The detection of partial subtype A1 or CRF02_AG sequences at these regions by SGS sequences also unequivocally confirmed that HIVE-hexahedron can reliably detect distinct viral populations that are missed by population-based consensus sequence methods.

**TABLE 4:** Comparison of recombination breakpoints determined by HIVE-hexahedron and Geneious-derived sequences for PK006^*a*^

HIVE-hexahedron		Genious	
Region	Subtype	Region	Subtype
Contig 1			
4905–6252	CRF02_AG	4906–6192	CRF02_AG
6253–7577	A1	6193–7516	A1
7578–8474	CRF02_AG	7577–7876	CRF02_AG
8475–8852	A1	7877–8176	A1
		8177–8965	CRF02_AG
8853–9624	CRF02_AG	8966–9606	A1
Contig 2			
5687–6253	CRF02_AG		
6254–6739	A1		
Contig 3			
6014–6604	CRF02_AG		
6605–7102	A1		
Contig 4			
6513–7055	A1		
Contig 5			
7578–7894	CRF02_AG		
7895–8078	A1		
8079–8879	CRF02_AG		
8880–9455	A1		
9456–9615	CRF02_AG		
Contig 6			
7779–7832	Indeterminable		
7833–8137	A1		
8138–8587	CRF02_AG		
8587–8694	A1		
Contig 7			
8879–9590	A1		
Contig 8			
8972–9624	A1		

a The shaded regions represent different recombination classifications determined with sequences generated by Geneious and HIVE-hexahedron.

Results similar to those observed in PK006 were obtained by analyzing an additional six viruses (Fig. S4 to S9). Multiple SGS sequences (21 to 59) were obtained from these six samples, and 1 to 7 viral populations were identified (Table 3). The complete major contig consensus sequence in 707010585 was very close to two highly similar SGS sequences (Fig. S4c), most likely due to the high similarity within the viral populations that allowed HIVE-hexahedron to easily assemble them together. Taken together, analysis of HTS reads from a long genome (>4,000 bp) by HIVE-hexahedron can identify distinct viral subpopulations when genetic diversity levels are high enough. These results show that all subpopulation sequences determined by HIVE-hexahedron represent either the viral populations (major or minor) at the region or the unique recombinant sequences, demonstrating that HIVE-hexahedron-derived sequences can represent actual viral sequences in the samples.

10.1128/mSphere.00551-20.4 FIG S4 Distinct viral populations determined in 707010585 by HIVE are validated by analysis of SGS-derived sequences. (a) Phylogenetic tree analysis and Highlighter plot of SGS-derived sequences from 707010585 identified four viral populations (A, B, C, and D; indicated by different-color bars). Phylogenetic trees were constructed using the neighbor-joining method and Kimura 2-parameter model. Highlighter plots show nucleotide substitutions compared to the SGS-derived sequence at the top and color-coded nucleotide substitutions. Each SGS-derived sequence is denoted by a symbol and corresponds to the horizontal lines on the Highlighter plot. The contig consensus sequences from HIVE-hexahedron analysis are included in the Highlighter plot for comparison. Three regions that are similar among all compared sequences (red boxes) were represented only in the main contig 1. (b) Sankey diagram showed five contigs: one major contig spanning the entire length of the 3′-half genome (1) and four smaller contigs (2 to 5). (c to g) Phylogenetic tree and Highlighter plot analysis of complete 3′-half genome sequences (c) and partial sequences (d to g). The relationship between HIVE-hexahedron contig sequences (red arrows) and SGS-derived sequences that overlap each other was determined individually by phylogenetic trees and Highlighter plots. Download FIG S4, PDF file, 1.0 MB. Copyright © 2020 Hora et al. 2020 Hora et al. This content is distributed under the terms of the Creative Commons Attribution 4.0 International license.

### Ambiguous bases can be resolved by parsing a quasispecies into distinct subpopulations using HIVE-hexahedron.

**FIG 7:**
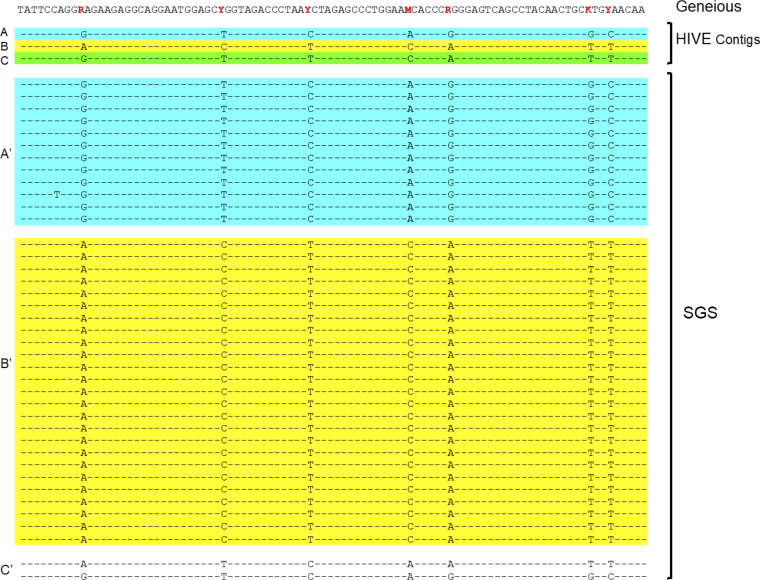
Ambiguous bases in the consensus sequence generated by Geneious are resolved by HIVE. Seven ambiguous bases in a 93-bp consensus sequence region generated by Geneious from PK006 are shown in red. All these ambiguous bases were clearly resolved among three viral populations (A in blue, B in yellow, and C in green) identified by HIVE using the same HTS data. Analysis of 39 SGS-derived sequences shows two major viral populations (A′ and B′, identical to the HIVE contig A and B sequences, respectively) and one minor population (C′) which is represented by only two sequences and does not represent any HIVE contig sequences.

Since HIVE-hexahedron can parse the quasispecies into distinct subpopulations, each subpopulation consensus would not be expected to have ambiguous bases. We next sought to determine how subpopulations identified by HIVE-hexahedron were able to resolve ambiguous bases in consensus sequences generated by Geneious using the SGS sequences generated for the same samples. For example, in a 93-bp region (nt 902 to 995) in the *vpr/tat* gene of PK006, the consensus sequence obtained by *de novo* assembly of raw reads using Geneious had seven ambiguous bases, indicating multiple bases were present at each of those sites (Fig. 7).

HIVE-hexahedron analysis of sequences in the same region identified three distinct viral populations (A, B, and C), with no ambiguous bases present in any consensus sequences for all populations. The analysis of 39 SGS sequences showed three subpopulations. All 12 SGS sequences, except one with an A/T mutation in subpopulations A′, were identical to the HIVE subpopulation A sequence. All 25 SGS sequences in subpopulation B′ were identical to the HIVE subpopulation B sequence. The other two SGS sequences constituted the last subpopulation C′, with the same bases at three sites and different bases at four other sites. Neither of them was the same as any HIVE subpopulation sequences. Thus, HIVE subpopulation C was not represented by the SGS sequences. This is most likely due to the limited SGS sequences available for analysis. Moreover, those two SGS sequences represent rare viral variants in the sample. These results demonstrate that HIVE-hexahedron can accurately detect different viral populations separately and generate consensus sequences without ambiguous bases for those subpopulations of HIV-1 quasispecies in a sample.

## DISCUSSION

Half (>4,000-bp) and complete (>9,000-bp) HIV-1 genomes have been increasingly analyzed by HTS. However, there are no computational algorithms that can reliably identify distinct subpopulation sequences generated by HTS from samples with quasispecies. Moreover, subsequent subtyping and recombination analyses require sequences to be exported and determined using additional softwares. To streamline all analyses in a single environment, we developed a new HIVE-hexahedron tool (32, 49, 50). After uploading the HTS raw reads in the HIVE platform, HIVE-hexahedron can identify different subpopulations, subtype all subpopulations, detect recombinant genomes, and determine recombinant breakpoints without exporting the sequences for further analysis.

When the short HTS raw reads from a sample with a complex HIV-1 quasispecies are analyzed, HIVE-hexahedron uses advanced algorithms to progressively align each read to multiple reference sequences. It will generate one major complete contig which represents either overall consensus of the viral population, clonally expanded viral population, or one of the viral subpopulations, while partial genomes where genetic diversity levels are higher than the preset threshold (1%) will be represented as subpopulations. Thus, the quasispecies in a sample will be parsed by subpopulations. The algorithm allows a user to select the threshold of the minimum accepted depth of coverage. In our study, we have selected the threshold of at least 50 reads. Therefore, the minimum sequencing depth required could be lower, but the user can select the threshold for confidence levels. Other tools, such as QuRe, ShoRah, and PredictHaplo, do not provide the granular option of selecting minimum coverage. However, due to the nature of the hexahedron algorithm, the main limiting factor in identifying a subpopulation is the length of the aligned read combined with the depth of coverage. Higher coverages (>1,000) and longer reads (>300 bp) will increase the likelihood to detect subpopulations. Thus, the detection of subpopulations in a sample will depend on both the depth of the sequence coverage and the sensitivity of the computer software algorithm (51). By comparing to SGS sequences, which characterize individual 3′-half genome sequences in one complete piece, we found that subpopulation sequences determined by HIVE-hexahedron were representative of distinct viral populations at the full 3′-half genome level or partial genome levels. Although the resolution of quasispecies determined by HIVE-hexahedron is not as high as that by SGS, the detection of subpopulations is a good indication of the presence of distinct viral subpopulations in a sample. Importantly, HIVE-hexahedron is much more cost-effective and faster than SGS. Moreover, each subpopulation sequence is clearly defined since a quasispecies is parsed into distinct subpopulations by HIVE-hexahedron. This may be particularly important when a drug-resistant quasispecies is analyzed since this can reveal if drug-resistant viruses were generated from the same or different subpopulations. These results demonstrate that HIVE-hexahedron is a powerful tool that can identify subpopulations in samples analyzed by short-read HTS. To our knowledge, this is the only algorithm with this capability and confidence level.

Unlike other computational algorithms, HIVE-hexahedron progressively aligns each read to multiple reference sequences. Thus, the HIVE-hexahedron pipeline allows us to rapidly determine HIV-1 subtypes by identifying the nearest neighbors (32, 49, 50) without the prior knowledge of the HIV-1 classification. The results are visualized as Sankey diagrams. The subtyping results of complete major population consensus sequences from 65 samples were confirmed by conventional subtype analysis of consensus sequences obtained by Geneious using standard HIV-1 subtyping tools. Importantly, the sequences in the reference set can be any number and exchanged for optimal alignment to identify the best-matched reference sequence. Thus, one of the unique features of HIVE-hexahedron is that it can automatically determine the subtype of each subpopulations.

Since subtypes of tested HIV-1 sequences can be easily determined using HIVE-hexahedron, the pipeline was optimized to detect recombinant sequences among different subtypes and define the recombination breakpoints. Using this pipeline, we found 10 of 65 viruses were recombinants, which were the same as those detected by Geneious-derived sequences. In eight samples, the recombination breakpoints determined by HIVE-hexahedron were similar to those determined by analyzing Geneious-derived sequences using the jpHHM and Simplot tools. In the last part of the 3′-half genomes of PK023 and PK006, the recombination breakpoints were different between the results from analysis of major population sequences generated by HIVE-hexahedron and Geneiouis. Analysis of subpopulation HIVE-hexahedron sequences showed that all recombination patterns identified by Geneious-derived sequences can be found in HIVE-hexahedron subpopulation sequences. This demonstrates that HIVE-hexahedron can accurately detect not only different coinfected HIV-1 strains but also distinct recombinant viral populations in the samples than the population sequencing-based methods like Geneious. The analysis of 39 SGS sequences from PK006 unequivocally confirmed the presence of distinct recombinant viral sequences. These results demonstrate that HIVE-hexahedron is powerful and accurate to determine coinfections and recombination patterns of a quasispecies.

Some HIVE subpopulations did not cluster with any major groups. They showed mosaic patterns among different subpopulations and could represent recombinant sequences. This might be because these recombinant populations identified by HIVE were unique and are not represented by limited SGS sequences. It is cautioned that recombination may be artificially generated during assembling of reads by HIVE-hexahedron because sequence diversity at some parts of the viral genome is too low and different subpopulations are genetically indistinguishable from each other. When HIVE-hexahedron progressively assembles the reads into contigs, it may switch between different viral subpopulations and generate artificial recombinant sequences. However, it is also possible that these recombinant sequences may be generated during PCR, which can result in high levels of PCR-mediated recombination (25, 63). Based on our previous study results (63), we used relatively low template input in each PCR to minimize the likelihood to generate PCR-mediated recombinants. However, this will not affect the ability of HIVE-hexahedron to detect different subpopulations at highly divergent subgenome regions. When HIVE-hexahedron was compared against other Quasispecies Spectrum Reconstruction (QSR) algorithms including QuRe, ShoRah, and PredictHaplo, it was found to either match or outperform the rest. In the *in silico* studies, type 2 error was less than the other QSR algorithms and in fact it was the only algorithm that, given enough depth of coverage and adequate length of the reads, was able to accurately reconstruct all subpopulations (51). HIVE-hexahedron should be further optimized to ensure that artificial recombination is minimized during assembly of short HTS reads. We plan to introduce a postcomputational step for statistical inference of potential artificial recombinants in HIVE-hexahedron to ensure that recombination is minimized in processing and assembly of short HTS reads.

The HIVE-hexahedron algorithm is powerful for streamline analysis of viral diversity, subtype, and recombination of short HTS data. It has a broad application by providing analysis of any quasispecies. In addition, HIVE as a platform can also be used for storing data and archiving results. This pipeline can be adapted readily to other less diverse RNA viruses (32).

## MATERIALS AND METHODS

### Plasma samples.

Plasma samples obtained from 33 HIV-1-infected individuals in a chronic infection cohort supported by the Centre for HIV/AIDS Vaccine Immunology (CHAVI) and from 32 HIV-1-infected individuals from a chronic infection cohort in Pakistan. All individuals were not treated with antiretroviral drugs. Written informed consent forms were obtained from all participants, and the studies were approved by the Duke University Institutional Review Board and the ethics committee of Bridge Consultants Foundation. Viral loads in these 65 individuals ranged from 610 to 760,000 copies per ml.

### Amplification of 3′-half HIV-1 genome.

Viral RNA was extracted from 400 μl of each plasma sample using EZ1 virus minikit v2.0 (Qiagen, Valencia, CA) and subjected to cDNA synthesis using Superscript III reverse transcriptase (Invitrogen, Carlsbad, CA) with primer 1.R3.B3R (5′-ACTACTTGAAGCACTCAAGGCAAGCTTTATTG-3′, HXB2, nt 9611 to 9642). The 3′-half genomes were amplified with undiluted cDNA from each sample in triplicate using Platinum *Taq* high-fidelity DNA polymerase (Life Technologies, Carlsbad, CA) as previously described (64). The primers for the first-round amplification were 07For7 (5′-CAAATTAYAAAAAATTCAAAATTTTCGGGTTTATTACAG-3′; nt 4875 to 4912) and 2.R3.B6R (5′-TGAAGCACTCAAGGCAAGCTTTATTGAGGC-3′; nt 9607 to 9636), and the primers for the second-round amplification were VIF1 (5′-GGGTTTATTACAGGGACAGCAGAG-3′; nt 4900 to 4923) and Low2C (5′-TGAGGCTTAAGCAGTGGGTTCC-3′; nt 9591 to 9612). The PCR thermocycling conditions were as follows: one cycle at 94°C for 2 minutes; 35 cycles of a denaturing step at 94°C for 15 seconds, an annealing step at 58°C for 30 seconds, and an extension step at 68°C for 5 minutes; and one cycle of an additional extension at 68°C for 10 minutes. Positive amplification was confirmed on an 0.8% agarose gel, and amplicons were subsequently purified using the AMPure XP beads (Beckman Coulter, Indianapolis, IN).

### High-throughput sequencing.

The purified PCR product (1 ng) from each sample was indexed to make the sequencing library (5 μl) using the Nextera XT kit (Illumina, San Diego, CA). The normalized libraries were pooled and further quantified by quantitative PCR (qPCR) using the KAPA library quantification kit (Kapa Biosystems, Woburn, MA). The libraries were sequenced using the MiSeq reagent kit v3 (2 × 150 cycles) on an Illumina MiSeq. Raw sequence reads were filtered for Q-scores above 30. The filtered high-quality sequences from each sample were parsed based on the unique sequence tags using “BaseSpace Sequence Hub” (Illumina, San Diego, CA). The sequences from both ends for the same cluster were paired and exported as Fastq files for subsequent analyses.

### Sequence assembling by Geneious.

Paired sequence reads from each sample were assembled using Geneious, a suite of the HTS analysis tools (Biomatters, Auckland, New Zealand) (65). A “map to reference” assembly was performed for each read pool using the Geneious assembler. The algorithm used is a seed and expand style mapper followed by an optional fine-tuning step to better align reads around indels to each other rather than the reference sequence. HXB2 was used as the reference to map the reads generated for each sample, and assembly was performed using a medium/fast sensitivity followed by fine-tuning of the assemblies with five times iteration. In this process, the highest-scoring sequence and its closest matching sequence are merged together into a contig. The consensus sequence for each sample was generated from the alignment of the reads and exported for subsequent analysis.

### Sequence assembling and analysis using HIVE-hexahedron.

The same paired end sequence reads used for analysis by Geneious were uploaded into HIVE. Additional quality control checking of raw reads such as average quality of bases per position was performed in HIVE (49, 50). In addition, reads were also examined for their quality based on average quality per read length and average quality per position. Only reads with average quality greater than a 30 Phred score were included for analysis.

After quality control of all reads, each paired-end sequence set was mapped to a set of reference sequences using the alignment tool, HIVE-hexagon (59). The parameters were set as default except some general and advanced parameters. To avoid the high variability among different HIV-1 subtype sequences and defective genes that are often detected in wild-type sequences, we used 20 consensus sequences of group M subtype (A1, A2, B, C, D, F1, G, and H), circulating recombinant forms (CRF_01, 02, 04, 06, 07, 08, 10, 11, 12, and 14), group O, and SIVcpz that were readily available from the Los Alamos HIV-1 sequence database (https://www.hiv.lanl.gov/). From the first HIVE-hexagon alignment, the references with reads per kilobase of transcript per million (RPKM) mapped value of 10,000 or more were selected as nearest neighbors for that sequence. To resolve the conflict for selection of one good reference among many closely related references, the analysis parameters used in our previous study (59) were modified. In addition, a specific algorithm that identified the nearest neighbor reference(s) to the raw reads from a pool of closely related references was implemented to HIVE-hexagon (59). The second alignment was done by aligning the raw reads from each sample to their nearest neighbor reference(s). The identified nearest neighbors were also indicative of the subtype of the sequence. If only one reference was the nearest neighbor, the sequence would represent the subtype of the sample. If there were more than one nearest neighbor, the individual was infected with different viruses or a recombinant between these nearest neighbors. For each sample with more than one identified nearest neighbor reference, multiple sequence alignments (MSAs) of nearest neighbor references were done using Multiple Alignment Fast Fourier Transform (MAFFT) (60) that was integrated in HIVE. The MSA of nearest neighbor references is used to create a common coordinate system for comparing the alignments of short reads from different references.

The final part of the pipeline is HIVE-hexahedron, a specialized tool in HIVE meant for reference-assisted *de novo* assembly of viral quasispecies (32). The input for HIVE-hexahedron is an alignment of sequences from a sample against its nearest neighbor reference sequence(s), and a multiple sequence alignment of the nearest neighbor reference sequences, in case there is more than one nearest neighbor. The output of HIVE-hexahedron is a Sankey diagram that uniquely shows if there are one or more viral populations in the sample and if recombinant genomes are present. It scans all variations above a given threshold in all available sequences and detects correlated groups of single nucleotide variation (SNV) patterns within each specific viral subpopulation. The subtypes and the recombination patterns are visualized using the interactive Sankey diagrams. Reads mapped against different sequences are remapped to a common coordinate system defined by the MSA (60) so they are piled up together. The consensuses of all subpopulation sequences can be exported in the FASTA format for further analysis. To improve the quality of the results, novel features were introduced in the tool that allow some postprocessing, which involves filtering out the small viral subpopulations with very low coverages. The following filters were used to define a subpopulation: at least 500 bp long, average depth of coverage of more than 50, and at least five SNVs.

### Sequence analysis.

The consensus sequences of viral subpopulations spanning the 3′-half genome (∼4,500 bp) of all samples were exported from Geneious and HIVE-hexahedron. The sequences were aligned together with references from the HIV Sequence Database using CLUSTAL W (66), and manual adjustments for optimal alignments were done using SEAVIEW. Phylogenetic trees were constructed using the neighbor-joining (NJ) method with the Kimura two-parameter model (67, 68), and the reliability of topologies was estimated by bootstrap analysis with 1,000 replicates. Recombination patterns were initially analyzed by REGA HIV-1 & 2 Automated Subtyping Tool (version 3.41; http://krisp.ukzn.ac.za/app/typingtool/virus/) and the jumping profile Hidden Markov Model (jpHMM) at the GOBICS website (http://jphmm.gobics.de/) (69). Highlighter plots were generated using the Highlighter tool at the Los Alamos HIV sequence database (https://www.hiv.lanl.gov/content/sequence/HIGHLIGHT/highlighter_top.html). The recombination breakpoints were confirmed by BootScan implemented in Simplot version 3.5.1 (70). The recombination pattern map for each viral subpopulation was generated using RecDraw (71).

### Single genome sequencing.

The cDNA was made by reverse transcription of viral RNA using primer 1.R3.B3R as described previously (64). The sequences of individual 3′-half genomes were obtained by single genome sequencing (SGS) from seven samples (26, 64). The first-round PCR primers were 2.R3.B6R and 07For7, and the second-round PCR primers were VIF1 and Low2C. The PCR products amplified from individual 3′-half genomes were purified and directly sequenced by HTS on an Illumina MiSeq.

### Accession number(s).

The GenBank accession numbers for sequences are MT395382 to MT395511 and MT419970 to MT420255.

10.1128/mSphere.00551-20.5 FIG S5 Distinct viral populations determined in 707010134 by HIVE are validated by analysis of SGS-derived sequences. (a) Phylogenetic tree analysis and Highlighter plot of SGS-derived sequences from 707010134 identified three viral populations (A, B, and C; indicated by different-color bars). Phylogenetic trees were constructed using the neighbor-joining method and Kimura 2-parameter model. Highlighter plots show nucleotide substitutions compared to the SGS-derived sequence at the top and color-coded nucleotide substitutions. Each SGS-derived sequence is denoted by a symbol and corresponds to the horizontal lines on the Highlighter plot. The contig consensus sequences from HIVE-hexahedron analysis are included in the Highlighter plot for comparison. (b) Sankey diagram showed three contigs: one major contig spanning the entire length of the 3′-half genome (1) and two smaller contigs (2 and 3). (c to e) Phylogenetic tree and Highlighter plot analysis of complete 3′-half genome sequences (c) and partial sequences (d and e). The relationship between HIVE-hexahedron contig sequences (red arrows) and SGS-derived sequences that overlap each other was determined individually by phylogenetic trees and Highlighter plots. Download FIG S5, PDF file, 1.2 MB. Copyright © 2020 Hora et al. 2020 Hora et al. This content is distributed under the terms of the Creative Commons Attribution 4.0 International license.

10.1128/mSphere.00551-20.6 FIG S6 Distinct viral populations determined in 707010038 by HIVE are validated by analysis of SGS-derived sequences. (a) Phylogenetic tree analysis and highlighter plot of SGS-derived sequences from 707010038 identified one viral population (indicated by black bar). Phylogenetic trees were constructed using the neighbor-joining method and Kimura 2-parameter model. Highlighter plots show nucleotide substitutions compared to the SGS-derived sequence at the top and color-coded nucleotide substitutions. Each SGS-derived sequence is denoted by a symbol and corresponds to the horizontal lines on the Highlighter plot. The contig consensus sequences from HIVE-hexahedron analysis are included in the Highlighter plot for comparison. (b) Sankey diagram showed three contigs: one major contig spanning the entire length of 3′-half genome (1) and two smaller contigs (2 and 3). (c to e) Phylogenetic tree and Highlighter plot analysis of complete 3′-half genome sequences (c) and partial sequences (d and e). The relationship between HIVE-hexahedron contig sequences (red arrows) and SGS-derived sequences that overlap each other was determined individually by phylogenetic trees and Highlighter plots. Download FIG S6, PDF file, 0.5 MB. Copyright © 2020 Hora et al. 2020 Hora et al. This content is distributed under the terms of the Creative Commons Attribution 4.0 International license.

10.1128/mSphere.00551-20.7 FIG S7 Distinct viral populations determined in PK013 by HIVE are validated by analysis of SGS-derived sequences. (a) Phylogenetic tree analysis and Highlighter plot of SGS-derived sequences from PK013 identified two viral populations (A and B; indicated by different-color bars). Phylogenetic trees were constructed using the neighbor-joining method and Kimura 2-parameter model. Highlighter plots show nucleotide substitutions compared to the SGS-derived sequence at the top and color-coded nucleotide substitutions. Each SGS-derived sequence is denoted by a symbol and corresponds to the horizontal lines on the Highlighter plot. The contig consensus sequences from HIVE-hexahedron analysis are included in the Highlighter plot for comparison. (b) Sankey diagram showed seven contigs: one major contig spanning the entire length of the 3′-half genome (1) and six smaller contigs (2 to 7). (c to i) Phylogenetic tree and Highlighter plot analysis of complete 3′-half genome sequences (c) and partial sequences (d to i). The relationship between HIVE-hexahedron contig sequences (red arrows) and SGS-derived sequences that overlap each other was determined individually by phylogenetic trees and Highlighter plots. Download FIG S7, PDF file, 1.3 MB. Copyright © 2020 Hora et al. 2020 Hora et al. This content is distributed under the terms of the Creative Commons Attribution 4.0 International license.

10.1128/mSphere.00551-20.8 FIG S8 Distinct viral populations determined in PK018 by HIVE are validated by analysis of SGS-derived sequences. (a) Phylogenetic tree analysis and Highlighter plot of SGS-derived sequences from PK018 identified four viral populations (A, B, and C [indicated by different-color bars] and recombinant sequences [green bars]). Phylogenetic trees were constructed using the neighbor-joining method and Kimura 2-parameter model. Highlighter plots show nucleotide substitutions compared to the SGS-derived sequence at the top, and color-coded nucleotide substitutions. Each SGS-derived sequence is denoted by a symbol and corresponds to the horizontal lines on the Highlighter plot. The contig consensus sequences from HIVE-hexahedron analysis are included in the Highlighter plot for comparison. The beginning region that was similar among all compared sequences (red box) was represented only in the main contig 1. (b) Sankey diagram showed five contigs: one major contig spanning the entire length of the 3′-half genome (1) and four smaller contigs (2 to 5). (c to g) Phylogenetic tree and Highlighter plot analysis of complete 3′-half genome sequences (c) and partial sequences (d to g). The relationship between HIVE-hexahedron contig sequences (red arrows) and SGS-derived sequences that overlap each other was determined individually by phylogenetic trees and Highlighter plots. Download FIG S8, PDF file, 1.1 MB. Copyright © 2020 Hora et al. 2020 Hora et al. This content is distributed under the terms of the Creative Commons Attribution 4.0 International license.

10.1128/mSphere.00551-20.9 FIG S9 Distinct viral populations determined in PK038 by HIVE are validated by analysis of SGS-derived sequences. (a) Phylogenetic tree analysis and Highlighter plot of SGS-derived sequences from PK038 identified three viral populations (A, B, and C; indicated by different-color bars). Phylogenetic trees were constructed using the neighbor-joining method and Kimura 2-parameter model. Highlighter plots show nucleotide substitutions compared to the SGS-derived sequence at the top and color-coded nucleotide substitutions. Each SGS-derived sequence is denoted by a symbol and corresponds to the horizontal lines on the Highlighter plot. The contig consensus sequences from HIVE-hexahedron analysis are included in the Highlighter plot for comparison. (b) Sankey diagram showed three contigs: one major contig spanning the entire length of the 3′-half genome (1) and two smaller contigs (2 and 3). (c to e) Phylogenetic tree and Highlighter plot analysis of complete 3′-half genome sequences (c) and partial sequences (d and e). The relationship between HIVE-hexahedron contig sequences (red arrows) and SGS-derived sequences that overlap each other was determined individually by phylogenetic trees and Highlighter plots. Download FIG S9, PDF file, 0.6 MB. Copyright © 2020 Hora et al. 2020 Hora et al. This content is distributed under the terms of the Creative Commons Attribution 4.0 International license.
