# Supplementary figures and images for: Streamlined Subpopulation, Subtype, and Recombination Analysis of HIV-1 Half-Genome Sequences Generated by High-Throughput Sequencing
Source: mSphere. 2020 Oct 14;5(5):e00551-20. doi: 10.1128/mSphere.00551-20 (PMC7565892; doi:10.1128/mSphere.00551-20)

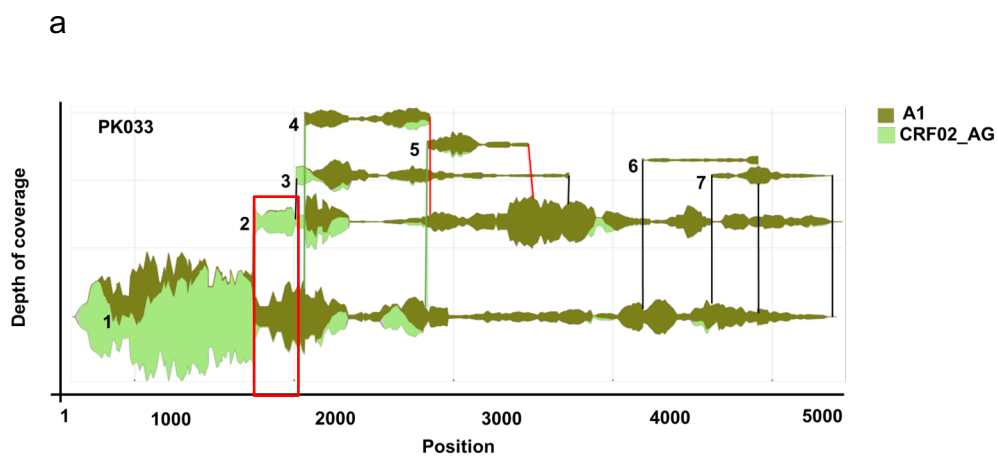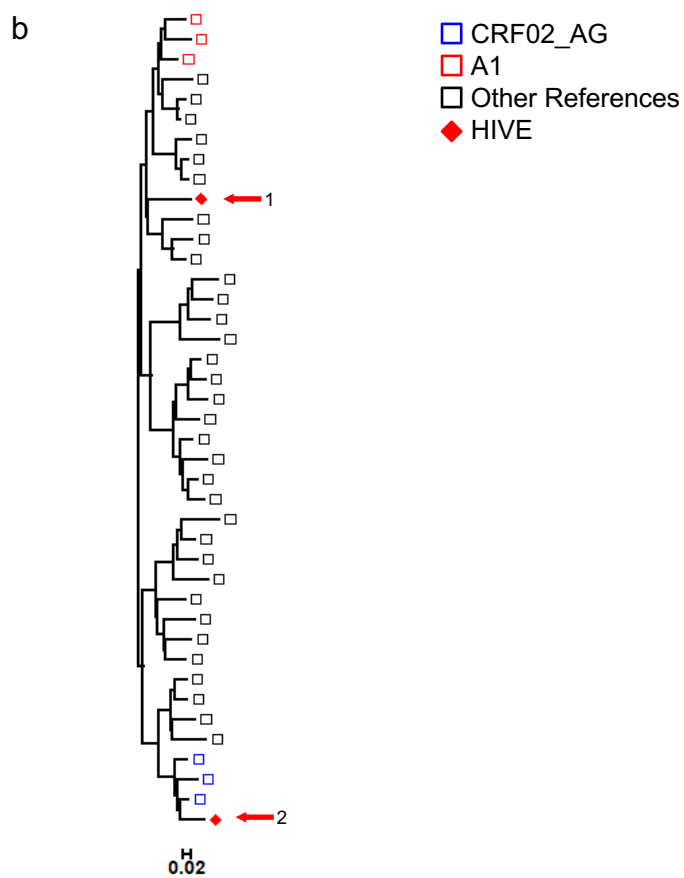

Fig. S2

Supplement: FIG S2 [file mSphere.00551-20-sf002.pdf]

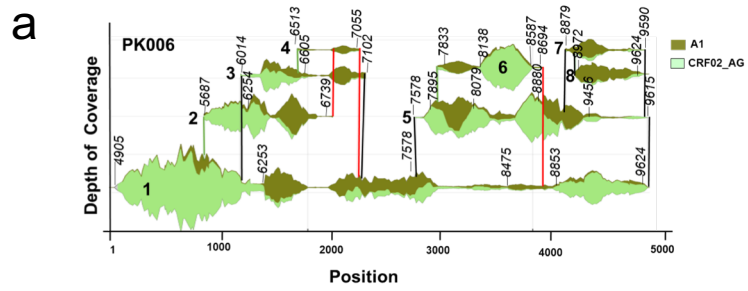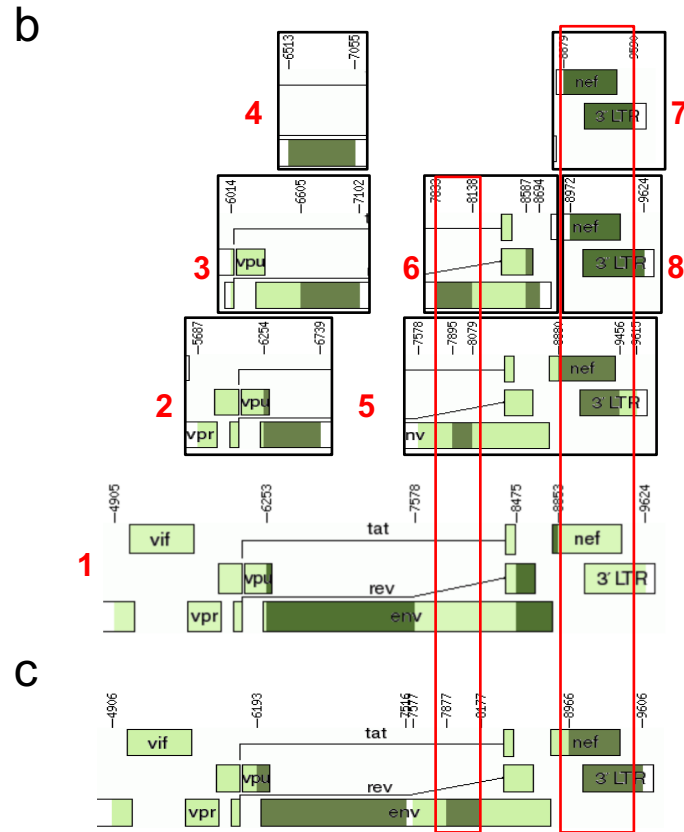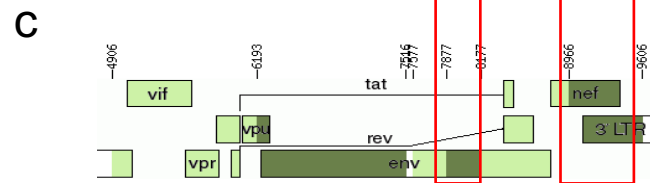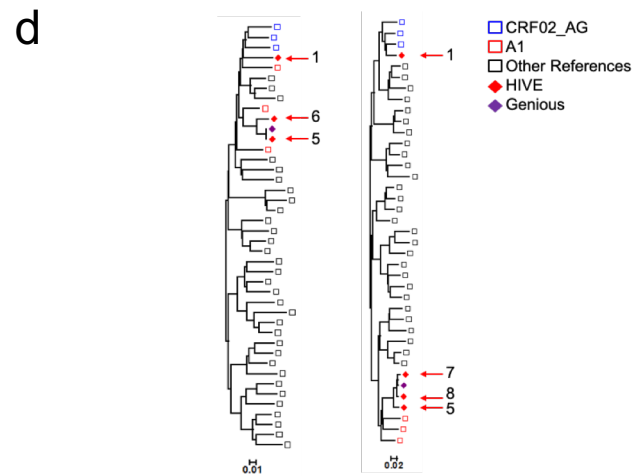

**Fig. S3**

Supplement: FIG S3 [file mSphere.00551-20-sf003.pdf]

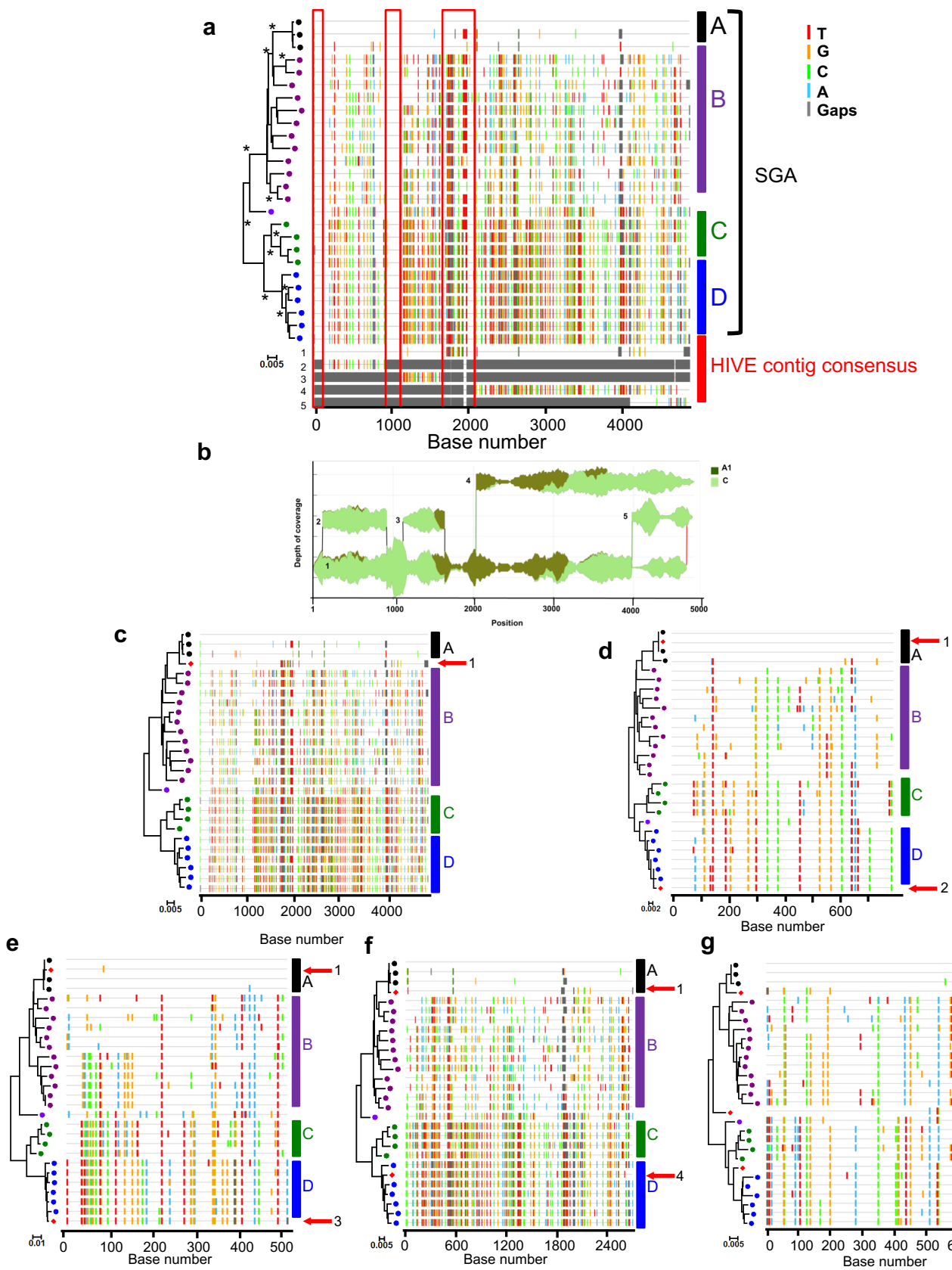

Supplement: FIG S4 [file mSphere.00551-20-sf004.pdf]

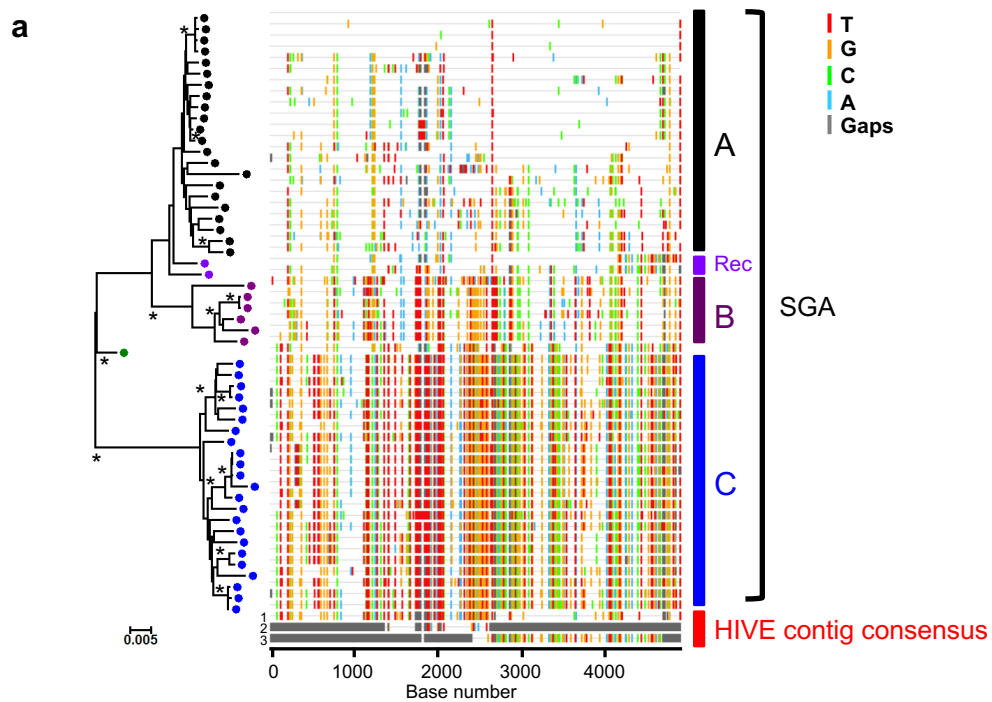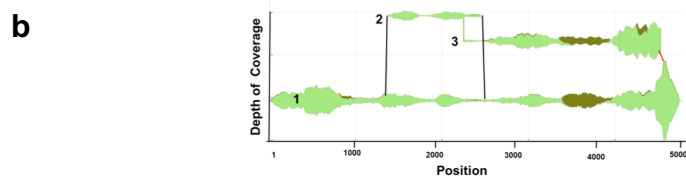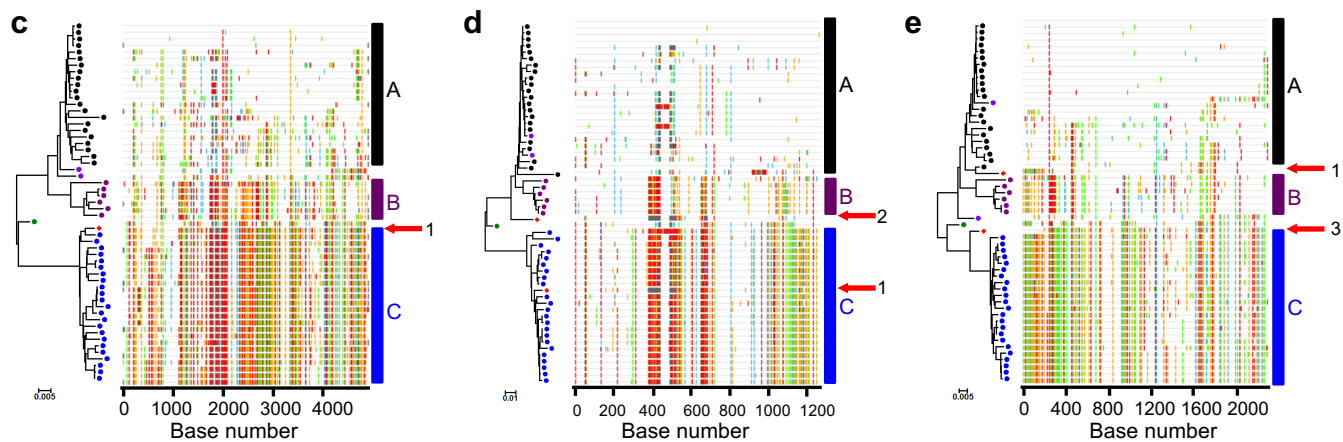

**Fig. S5**

Supplement: FIG S5 [file mSphere.00551-20-sf005.pdf]

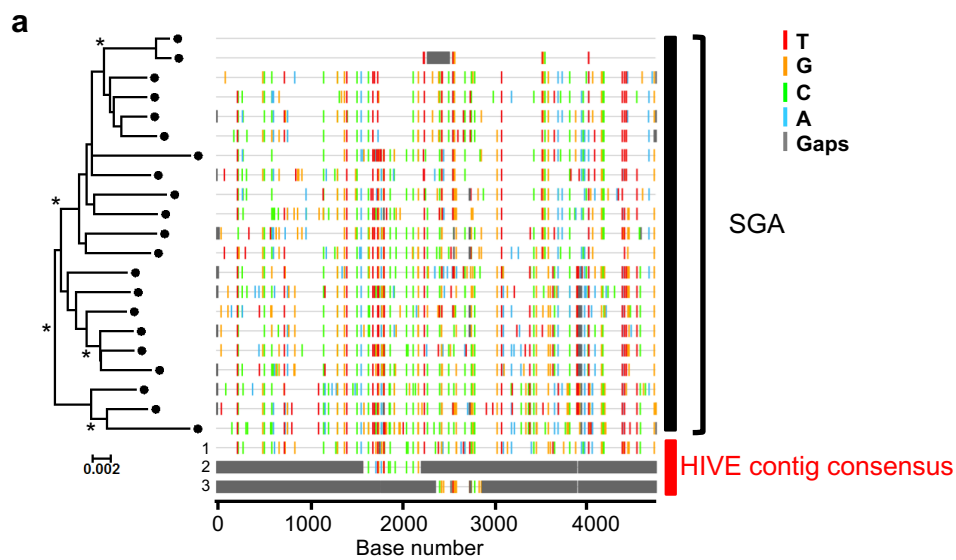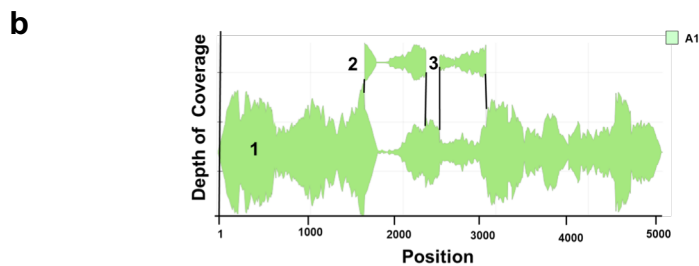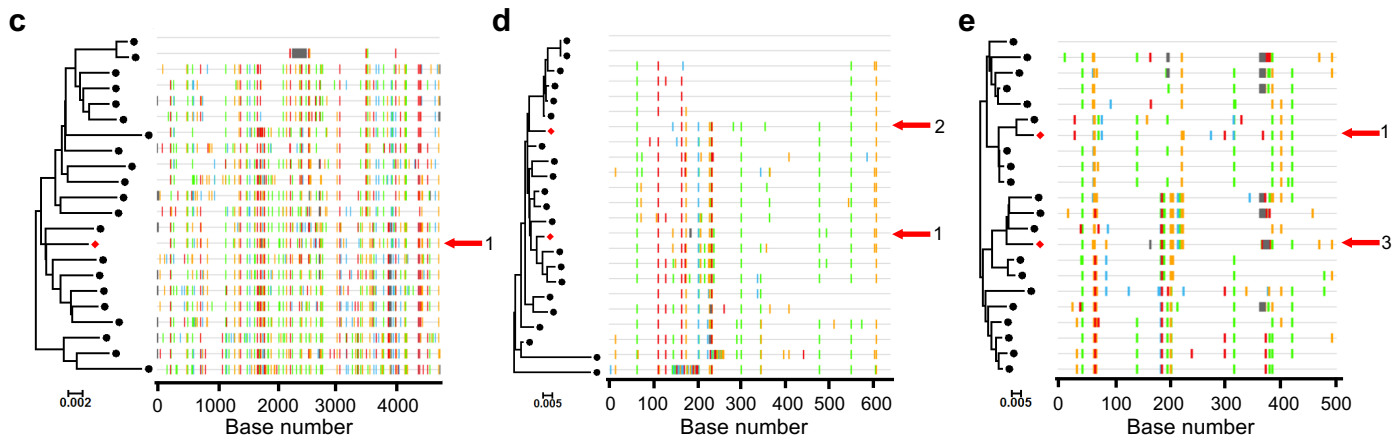

**Fig. S6**

Supplement: FIG S6 [file mSphere.00551-20-sf006.pdf]

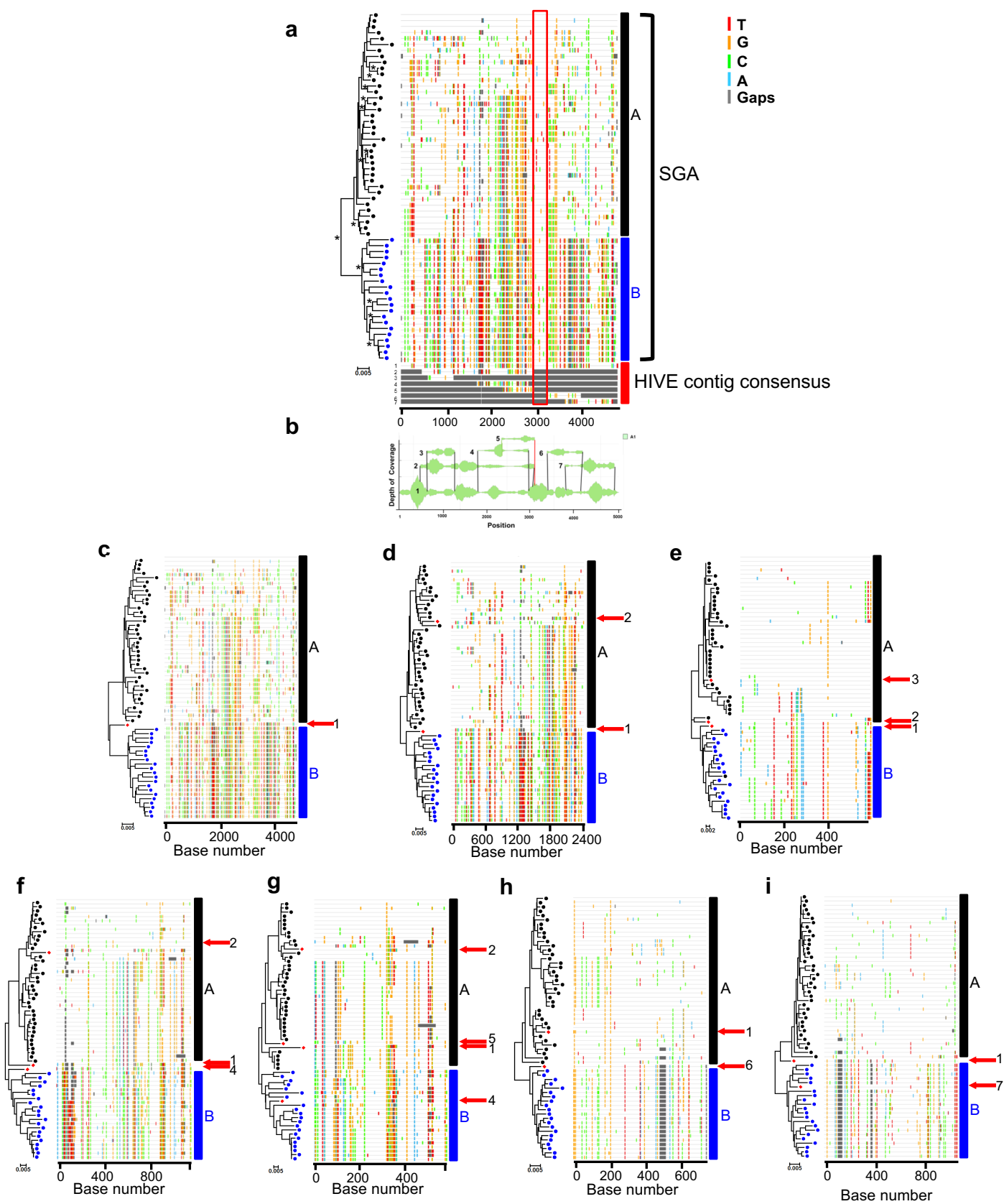

**Fig. S7**

Supplement: FIG S7 [file mSphere.00551-20-sf007.pdf]

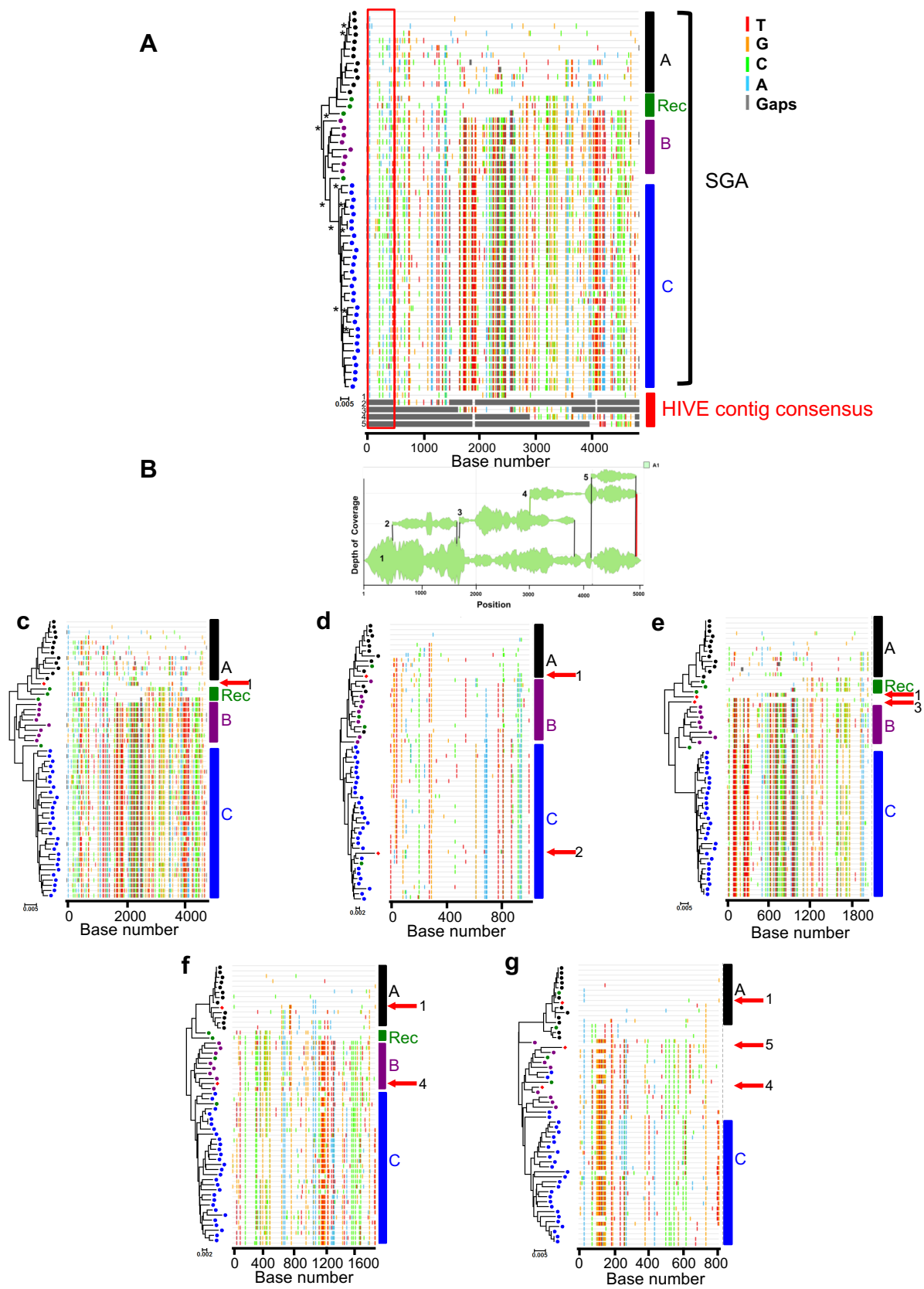

**Fig. S8**

Supplement: FIG S8 [file mSphere.00551-20-sf008.pdf]

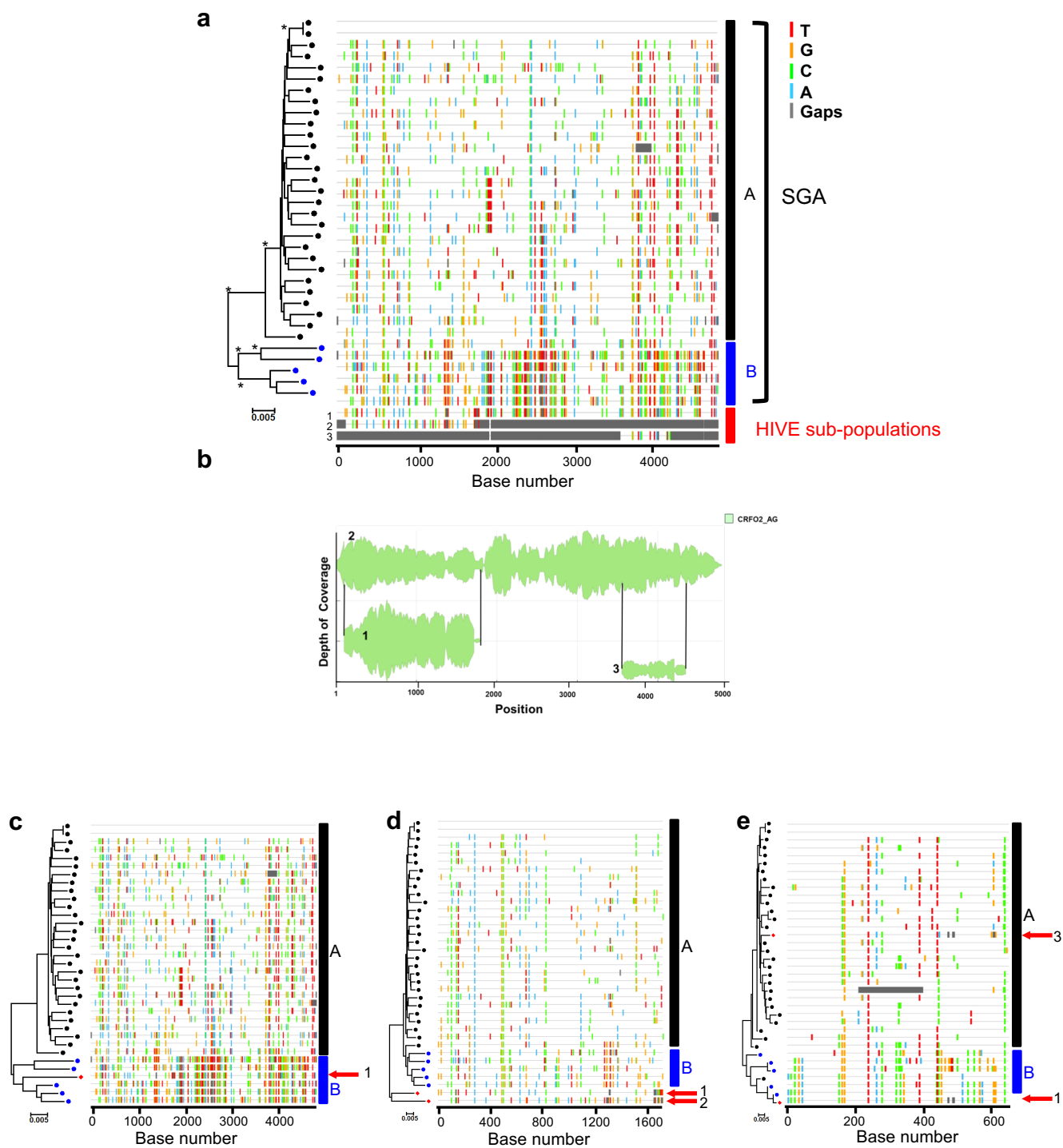

**Fig. S9**

Supplement: FIG S9 [file mSphere.00551-20-sf009.pdf]
