# Supplementary material for: Streamlined Subpopulation, Subtype, and Recombination Analysis of HIV-1 Half-Genome Sequences Generated by High-Throughput Sequencing
Source: mSphere. 2020 Oct 14;5(5):e00551-20. doi: 10.1128/mSphere.00551-20 (PMC7565892; doi:10.1128/mSphere.00551-20)
Supplement: FIG S1 [file mSphere.00551-20-sf001.pdf]

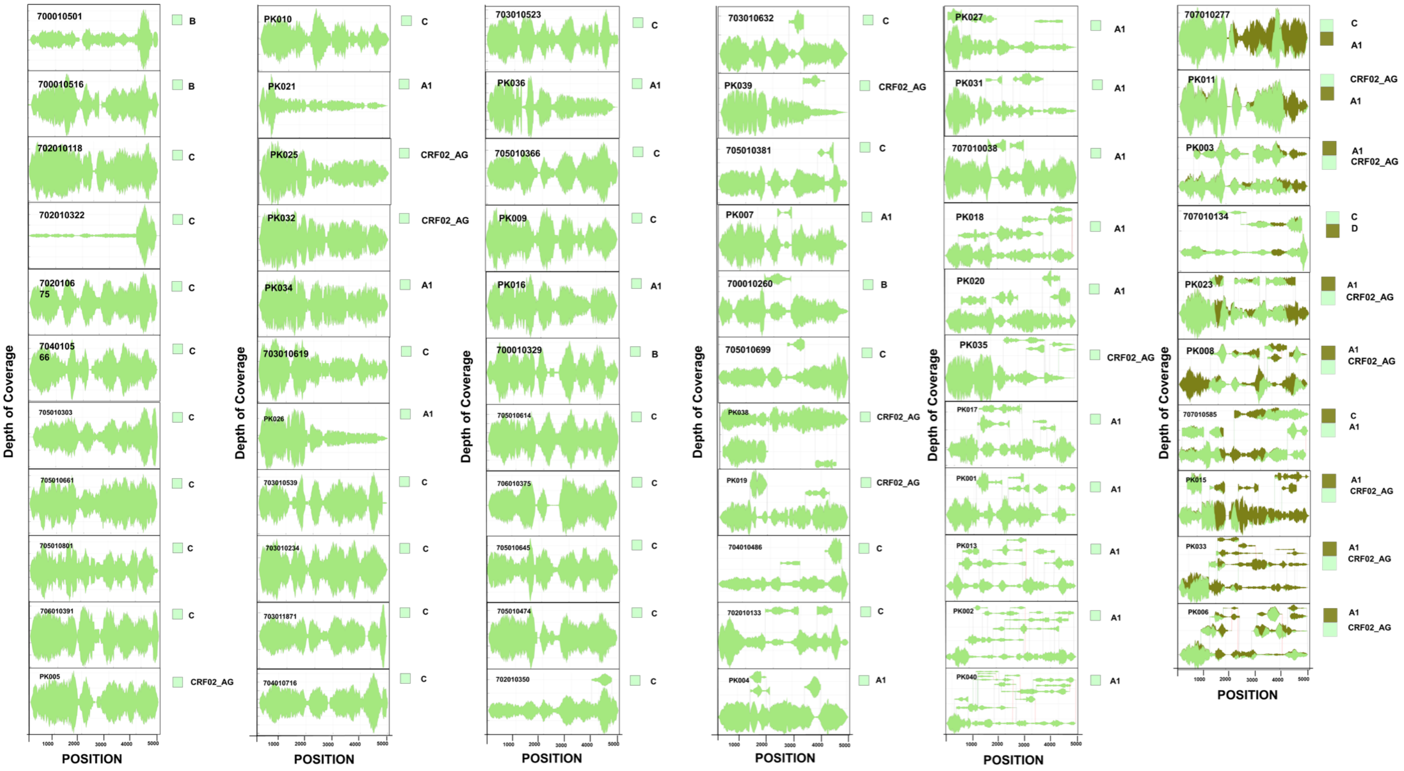

Depth of Coverage
POSITION
700010501
B
700010516
B
702010118
C
702010322
C
7020106 75
C
7040105 66
C
705010303
C
705010661
C
705010801
C
706010391
C
PK005
CRF02_AG
PK010
C
PK021
A1
PK025
CRF02_AG
PK032
CRF02_AG
PK034
A1
703010619
C
PK026
A1
703010539
C
703010234
C
703011871
C
704010716
C
703010523
C
PK036
A1
705010366
C
PK009
C
PK016
A1
700010329
B
705010614
C
706010375
C
705010645
C
705010474
C
702010350
C
703010632
C
PK039
CRF02_AG
705010381
C
PK007
A1
700010260
B
705010699
C
PK038
CRF02_AG
PK019
CRF02_AG
704010486
C
702010133
C
PK004
A1
PK027
A1
PK031
A1
707010038
A1
PK018
A1
PK020
A1
PK035
CRF02_AG
PK017
A1
PK001
A1
PK013
A1
PK002
A1
PK040
A1
707010277
C
A1
PK011
CRF02_AG
A1
PK003
A1
CRF02_AG
707010134
C
D
PK023
A1
CRF02_AG
PK008
A1
CRF02_AG
707010585
C
A1
PK015
A1
CRF02_AG
PK033
A1
CRF02_AG
PK006
A1
CRF02_AG
1000
2000
3000
4000
5000

Fig. S1
